# Disparities in Cancer Mortality among Disaggregated Asian American Subpopulations, 2018–2021

**DOI:** 10.1007/s40615-024-02067-0

**Published:** 2024-06-25

**Authors:** David T. Zhu, Alan Lai, Andrew Park, Anthony Zhong, Suzanne Tamang

**Affiliations:** 1 Medical Scientist Training Program, School of Medicine, Virginia Commonwealth University, Richmond, VA 23298 USA; 2 School of Medicine, Virginia Commonwealth University, 1201 E Marshall St, Richmond, VA 23298 USA; 3 Harvard Medical School, Boston, MA 02115 USA; 4 Department of Medicine, Stanford University School of Medicine, Stanford, CA 94305 USA; 5 Program Evaluation Resource Center, Office of Mental Health and Suicide Prevention, Department of Veterans Affairs, Menlo Park, CA 94025 USA

**Keywords:** Cancer, Mortality, Asian American, Disaggregated

## Abstract

Federal, state, and institutional data collection practices and analyses involving Asian Americans as a single, aggregated group obscure critical health disparities among the vast diversity of Asian American subpopulations. Using from the Centers for Disease Control and Prevention Wide-Ranging Online Data for Epidemiologic Research (CDC WONDER) Underlying Causes of Death database, we conducted a cross-sectional study using data on disaggregated Asian American subgroups (Asian Indian, Chinese, Filipino, Japanese, Korean, Vietnamese, other Asians) between 2018 and 2021. We examine deaths from 22 cancer types and in situ, benign neoplasms, identified using ICD-10 codes C00-C97 and D00-D48. Overall, our study comprised 327,311 Asian American decedents, with a mean age of death at 70.57 years (SD=2.79), wherein females accounted for approximately half of the sample (*n*=36,596/73,207; 49.99%). Notably, compared to the aggregated Asian American reference group, we found higher proportions of deaths from total cancers among Chinese (25.99% vs. 22.37% [ref]), Korean (25.29% vs. 22.37% [ref]), and Vietnamese (24.98% vs. 22.37% [ref]) subgroups. In contrast, total cancer deaths were less prevalent among Asian Indians (17.49% vs. 22.37% [ref]), Japanese (18.90% vs. 22.37% [ref]), and other Asians (20.37% vs. 22.37% [ref]). We identified further disparities by cancer type, sex, and age. Disaggregated data collection and analyses are imperative to understanding differences in cancer mortality among Asian American subgroups, illustrating at-risk populations with greater granularity. Future studies should aim to describe the association between these trends and social, demographic, and environmental risk factors.

**Supplementary Information:**

The online version contains supplementary material available at 10.1007/s40615-024-02067-0.

## Introduction

Asian Americans, Native Hawaiian, and Pacific Islanders (AANHPI) are one of the fastest growing racial and ethnic groups in the United States (US), accounting for 20.6 million individuals (6% of total US population) and are projected to rise to 9% of the total US population by 2060 [1–3]. Unlike all other racial and ethnic groups — for whom heart disease is the leading cause of death — cancer has been the leading cause of death for AANHPI individuals since the early 2000s. [4–7] As AANHPI immigration increases and the older population size expands [3], the AANHPI cancer burden may continue to increase.

Despite remarkable heterogeneity, with over 50 different AANHPI subgroups widely distributed across different ethno-geographic regions in the US, AANHPI populations are often categorized as a single monolithic population within local and national health databases [4, 8]. Often described as a “model minority,” AANHPI communities are subjected to a myth that they uniformly enjoy better health than other populations due to academic and economic success [1]. The roots of such generalizations can be traced back to the 1985 Heckler Report asserting “[t]he Asian/Pacific Island minority, in aggregate, is healthier than all racial/ethnic groups in the United States, including Whites” [9, 10]. Thus, prior findings that aggregated Asian Americans are at a lower risk of cancer mortality compared to non-Hispanic White populations overlook the intricate diversity within various Asian communities, blurring critical cancer disparities between subgroups and variations in cancer subtypes, geographic origin, immigration, acculturation, dietary factors, and socioeconomic status [9–11]. For instance, previous studies have shown significant differences in the rates of age-standardized mortality due to various cancers and cancer screening between disaggregated Asian American subgroups [12, 13].

Additionally, prior research highlights significant age and gender disparities. For instance, while both Asian American men and women exhibit higher rates of liver and intrahepatic bile duct cancers compared to their White counterparts, the incidence and mortality rates are more than double in Asian American men compared to women [14]. This disparity may be attributed to a complex interplay of genetic, physiological, psychological, behavioral, and environmental risk factors. For example, Asian American men typically have higher levels of androgens, alcohol consumption, tobacco use, and a greater prevalence of chronic hepatitis B infections compared to Asian American women [7, 15], potentially increasing their risk of developing liver cancer or experiencing worse prognoses.

Moreover, previous studies highlight that there are significant age-related disparities. Asian Americans aged 35–49 years and those over 65 years have significantly higher incidence rates of hepatocellular carcinoma (HCC) compared to Hispanic populations, while Asian Americans aged 50–64 years have relatively lower HCC incidence rates compared to their Hispanic counterparts [16]. To ensure the effectiveness of clinical and public health interventions, it is crucial to adapt these interventions to the unique needs of different gender and age groups, incorporating culturally-tailored approaches.

Federal US agencies are currently only mandated to adhere to minimum reporting standards set by the Office of Management and Budget (OMB), limiting classifications to broad categories like “Asian” and “Native Hawaiian or Other Pacific Islander”, which have remained unchanged since 1997 [17]. With the omission of disaggregated racial and ethnic data reporting for Asian American subgroups, analyses may lack sufficient granularity required for effective health interventions, identifying at-risk groups, and shaping health policy priorities [18, 19].

Previous studies have comprehensively assessed disparities in mortality from various cancer types across disaggregated Asian American subgroups, typically comparing them to the general population or non-Hispanic White population [20, 21]. However, to our knowledge, no studies have compared cancer mortality between disaggregated Asian American subgroups and the aggregated Asian American population. This comparison is crucial to investigate how disparities may be obscured by using the overarching term “Asian American” rather than assessing specific subgroups. Thus, to better understand disparities in cancer-specific mortality rates between different Asian American ethnic groups, we conducted a comprehensive analysis of 22 different cancers and neoplasms using disaggregated data, ultimately aiming to inform future cancer screening programs and clinical or public health interventions.

## Methods

Using data from the Centers for Disease Control and Prevention Wide-Ranging Online Data for Epidemiologic Research (CDC WONDER), this cross-sectional study examined death certificates among Asian American individuals for various cancers/neoplasms. We adhered to Strengthening the Reporting of Observational Studies in Epidemiology (STROBE) reporting guidelines for cross-sectional studies (Table S1). Due to the publicly available, deidentified nature of the data, institutional review was not sought from Virginia Commonwealth University and informed consent were not applicable, following HHS regulation 45 CFR 46.101(c).

Specifically, we examined death certificates for 22 types of neoplasms as the underlying cause of death between 2018 (the earliest year in which disaggregated racial and ethnic data were made available for Asian American populations in CDC WONDER) to 2021, identified using the following International Classification of Diseases, Tenth Revision (ICD-10) codes. These ICD-10 codes were as follows: C00-C97 (total cancers); C00-C14 (lip, oral, and pharyngeal cancers); C15 (esophageal cancer); C16 (stomach cancer); C18-C21 (colorectal and anal cancers); C22 (liver and intrahepatic bile duct cancers); C25 (pancreatic cancer); C32 (laryngeal cancer); C33-C34 (tracheal, bronchial, and lung cancers); C43 (skin cancer); C50 (breast cancers); C53-C55 (cervical and uterine cancers); C56 (ovarian cancer); C61 (prostate cancer); C64-C65 (kidney cancer); C67 (bladder cancer); C70-C72 (brain, meningeal, or other central nervous system cancers); C81 (Hodgkin disease); C82-C85 (non-Hodgkin lymphoma); C91-C95 (leukemia); C88, C90 (multiple myeloma and immunoproliferative neoplasms); C17, C23-C24, C26-C31, C37-C41, C44-C49, C51-C52, C57-C60, C62-C63, C66, C68-C69, C73-C80, C97 (other and unspecified cancers); and D00-D48 (neoplasms in situ, benign, or of uncertain or unknown behavior).

### Statistical Analysis

We calculated crude, sex-stratified, and age-stratified (<35, 35–44, 45–54, 55–64, 65–74, 75–84, 85+years) proportional mortality values for each cancer/neoplasm within disaggregated Asian American subgroups (Asian Indian, Chinese, Filipino, Japanese, Korean, Vietnamese, other Asians), in comparison to the aggregated Asian American reference group. Notably, the CDC WONDER database categorized all decedents not classified into any of the six specific disaggregated ethnic groups as 'Other Asians'. We also calculated the mean age at death from each type of neoplasm for each racial and ethnic group. We used R (version 4.3.0) to perform two proportion Z-tests to identify significant differences at α=0.05. Considering substantial levels of data suppression for NHPI populations, we omitted these data from statistical analyses. Instead, we included these raw data in (Tables S2-S3).

## Results

Our diverse study sample comprised 327,311 Asian American decedents. Within the aggregated Asian American group, 73,207 individuals (22.37%) died from cancer, with a mean age of death of 70.57 (SD=2.79) years. Females comprised half of the sample (*n*=36,596/73,207; 49.99%) of cancer deaths. The five most common types of cancers were tracheal, bronchial, and lung cancers (*n*=14,726/73,207; 20.12%); other and unspecified cancers (*n*=8,797/73,207; 12.02%); colorectal cancer (*n*=7,325/73,207; 10.01%); liver and intrahepatic bile duct cancers (*n*=6,626/73,207; 9.05%); and pancreatic cancer (*n*=6,065//73,207; 8.28%) (Table 1).

**Table 1:** Asian-American digestive and respiratory cancer deaths, 2018–2021

Cause of death	Aggregate Asian	Asian Indian	Chinese	Filipino	Japanese	Korean	Vietnamese	Other Asian
Total decedents	327,311 (100.00)	45,133 (100.00)	73,051 (100.00)	65,107 (100.00)	36,331 (100.00)	28,983 (100.00)	33,055 (100.00)	45,651 (100.00)
Total cancers								
Overall (N, %)	73,207 (22.37)	7,892 (17.49)***	18,987 (25.99)***	14,571 (22.38)	6,868 (18.90)***	7,330 (25.29)***	8,258 (24.98)***	9,301 (20.37)***
Mean age of death (years, SD)	70.62 (0.77)	69.10 (0.81)	72.33 (0.79)	70.57 (0.83)	78.49 (1.03)	72.34 (0.88)	68.59 (0.76)	66.60 (0.72)
Sex								
Female (*N*, %)	36,596 (11.18)	3,541 (7.85)***	9,297 (12.73)***	8,036 (12.34)***	4,037 (11.11)	3,866 (13.34)***	3,289 (9.95)***	4,530 (9.92)***
Male (*N*, %)	36,611 (11.19)	4,351 (9.64)***	9,690 (13.26)***	6,535 (10.04)***	2,831 (7.79)***	3,464 (11.95)***	4,969 (15.03)***	4,771 (10.45)***
Suppressed or sex not stated (*N*, %)	0 (0.00)	0 (0.00)	0 (0.00)	0 (0.00)	0 (0.00)	0 (0.00)	0 (0.00)	0 (0.00)
Age								
< 35 years (*N*, %)	1,182 (0.36)	219 (0.49)***	253 (0.35)	160 (0.25)***	13 (0.04)***	44 (0.15)***	119 (0.36)	315 (0.69)***
35–44 years (*N*, %)	2,127 (0.65)	319 (0.71)	483 (0.66)	401 (0.62)	63 (0.17)***	157 (0.54)*	251 (0.76)*	453 (0.99)***
45–54 years (*N*, %)	6,221 (1.90)	733 (1.62)***	1,499 (2.05)**	1,200 (1.84)	203 (0.56)***	539 (1.86)	937 (2.83)***	1,110 (2.43)***
55–64 years (*N*, %)	13,239 (4.04)	1,447 (3.21)***	3,212 (4.40)***	2,674 (4.11)	778 (2.14)***	1,245 (4.30)*	1,835 (5.55)***	2,048 (4.49)***
65–74 years (*N*, %)	19,126 (5.84)	2,178 (4.83)***	4,671 (6.39)***	4,175 (6.41)***	1,434 (3.95)***	1,804 (6.22)**	2,265 (6.85)***	2,599 (5.69)
75–84 years (*N*, %)	18,512 (5.66)	2,048 (4.54)***	4,648 (6.36)***	3,993 (6.13)***	1,708 (4.70)***	2,316 (7.99)***	1,893 (5.73)	1,906 (4.18)***
≥ 85 years (*N*, %)	12,800 (3.91)	946 (2.10)***	4,215 (5.77)***	1,961 (3.01)***	2,654 (7.31)***	1,203 (4.15)*	955 (2.89)***	866 (1.90)***
Suppressed or age not stated (N, %)	0 (0.00)	2 (0.00)	6 (0.01)	7 (0.01)	15 (0.04)	22 (0.08)	3 (0.01)	4 (0.01)
Lip, oral, and pharyngeal cancers								
Overall (N, %)	1,655 (0.51)	326 (0.72)***	498 (0.68)***	225 (0.35)***	95 (0.26)***	83 (0.29)***	180 (0.54)	248 (0.54)
Mean age of death (years, SD)	67.75 (0.75)	64.58 (1.86)	67.51 (1.36)	72.17 (2.96)	—	—	—	66.57 (2.67)
Sex								
Female (*N*, %)	535 (0.16)	83 (0.18)	151 (0.21)*	91 (0.14)	46 (0.13)	29 (0.10)*	48 (0.15)	87 (0.19)
Male (*N*, %)	1,120 (0.34)	243 (0.54)***	347 (0.48)***	134 (0.21)***	49 (0.13)***	54 (0.19)***	132 (0.40)	161 (0.35)
Suppressed or sex not stated (N, %)	0 (0.00)	0 (0.00)	0 (0.00)	0 (0.00)	0 (0.00)	0 (0.00)	0 (0.00)	0 (0.00)
Age								
< 35 years (*N*, %)	24 (0.01)	—	—	—	—	—	—	—
35–44 years (*N*, %)	79 (0.02)	15 (0.03)	29 (0.04)*	—	—	—	—	13 (0.03)
45–54 years (*N*, %)	221 (0.07)	42 (0.09)	79 (0.11)***	16 (0.02)***	—	—	37 (0.11)	37 (0.08)
55–64 years (*N*, %)	396 (0.12)	93 (0.21)***	109 (0.15)	43 (0.07)***	—	18 (0.06)**	51 (0.15)	75 (0.16)*
65–74 years (*N*, %)	401 (0.12)	83 (0.18)***	119 (0.16)**	66 (0.10)	23 (0.06)**	18 (0.06)**	41 (0.12)	51 (0.11)
75–84 years (*N*, %)	308 (0.09)	64 (0.14)**	91 (0.12)*	49 (0.08)	23 (0.06)	22 (0.08)	24 (0.07)	35 (0.08)
≥ 85 years (*N*, %)	221 (0.07)	26 (0.06)	64 (0.09)	38 (0.06)	36 (0.10)*	14 (0.05)	16 (0.05)	27 (0.06)
Suppressed or age not stated (*N*, %)	5 (0.00)	3 (0.01)	7 (0.01)	13 (0.02)	13 (0.04)	11 (0.04)	11 (0.03)	10 (0.02)
Esophageal cancers								
Overall (*N*, %)	1,238 (0.38)	206 (0.46)*	291 (0.40)	154 (0.24)***	169 (0.47)*	93 (0.32)	144 (0.44)	181 (0.40)
Mean age of death (years, SD)	70.55 (0.94)	72.58 (4.56)	72.57 (2.35)	72.00 (7.16)	74.36 (2.67)	—	—	—
Sex								
Female (*N*, %)	312 (0.10)	67 (0.15)**	67 (0.09)	26 (0.04)***	59 (0.16) ***	22 (0.08)	11 (0.03)***	60 (0.13)*
Male (*N*, %)	926 (0.28)	139 (0.31)	224 (0.31)	128 (0.20)	110 (0.30)**	71 (0.24)	133 (0.40)***	121 (0.27)
Suppressed or sex not stated (*N*, %)	0 (0.00)	0 (0.00)	0 (0.00)	0 (0.00)	0 (0.00)	0 (0.00)	0 (0.00)	0 (0.00)
Age								
< 35 years (*N*, %)	—	—	—	—	—	—	—	—
35–44 years (*N*, %)	24 (0.01)	—	—	—	—	—	—	—
45–54 years (*N*, %)	97 (0.03)	17 (0.04)	17 (0.02)	16 (0.02)	—	—	16 (0.05)	16 (0.04)
55–64 years (*N*, %)	283 (0.09)	34 (0.08)	58 (0.08)	45 (0.07)	33 (0.09)	12 (0.04)*	50 (0.15)***	51 (0.11)
65–74 years (*N*, %)	389 (0.12)	69 (0.15)	85 (0.12)	51 (0.08)**	51 (0.14)	30 (0.10)	49 (0.15)	54 (0.12)
75–84 years (*N*, %)	286 (0.09)	52 (0.12)	81 (0.11)	28 (0.04)***	43 (0.12)	29 (0.10)	16 (0.05)*	37 (0.08)
≥ 85 years (*N*, %)	151 (0.05)	27 (0.06)	47 (0.06)	—	35 (0.10)***	11 (0.04)	—	14 (0.03)
Suppressed or age not stated (*N*, %)	8 (0.00)	7 (0.02)	3 (0.00)	14 (0.02)	7 (0.02)	11 (0.04)	13 (0.04)	9 (0.02)
Stomach cancers								
Overall (*N*, %)	3,399 (1.04)	202 (0.45)***	1,043 (1.43)***	346 (0.53)***	325 (0.89)*	650 (2.24)***	369 (1.12)	464 (1.02)
Mean age of death (years, SD)	70.31 (3.72)	66.45 (3.17)	71.85 (3.95)	68.81 (3.53)	80.66 (5.69)	73.76 (1.14)	67.35 (3.21)	66.44 (3.19)
Sex								
Female (*N*, %)	1,544 (0.47)	75 (0.17)***	464 (0.64)***	182 (0.28)***	190 (0.52)	288 (0.99)***	143 (0.43)	202 (0.44)
Male (*N*, %)	1,855 (0.57)	127 (0.28)***	579 (0.79)***	164 (0.25)***	135 (0.37)***	362 (1.25)***	226 (0.68)**	262 (0.57)
Suppressed or sex not stated (*N*, %)	0 (0.00)	0 (0.00)	0 (0.00)	0 (0.00)	0 (0.00)	0 (0.00)	0 (0.00)	0 (0.00)
Age								
< 35 years (*N*, %)	57 (0.02)	—	15 (0.02)	—	—	—	—	16 (0.04)*
35–44 years (*N*, %)	124 (0.04)	17 (0.04)	31 (0.04)	—	—	14 (0.05)	20 (0.06)	30 (0.07)**
45–54 years (*N*, %)	332 (0.10)	33 (0.07)	76 (0.10)	53 (0.08)	12 (0.03)***	44 (0.15)*	48 (0.15)*	66 (0.14)*
55–64 years (*N*, %)	575 (0.18)	33 (0.07)***	178 (0.24)***	56 (0.09)***	29 (0.08)***	124 (0.43)***	68 (0.21)	87 (0.19)
65–74 years (*N*, %)	860 (0.26)	38 (0.08)***	261 (0.36)***	103 (0.16)***	64 (0.18)**	159 (0.55)***	107 (0.32)*	128 (0.28)
75–84 years (*N*, %)	832 (0.25)	54 (0.12)***	263 (0.36)***	80 (0.12)***	74 (0.20)	194 (0.67)***	74 (0.22)	93 (0.20)*
≥ 85 years (*N*, %)	613 (0.19)	19 (0.04)***	218 (0.30)***	36 (0.06)***	142 (0.39)***	111 (0.38)***	46 (0.14)	41 (0.09)***
Suppressed or age not stated (*N*, %)	6 (0.00)	8 (0.02)	1 (0.00)	18 (0.03)	4 (0.01)	4 (0.01)	6 (0.02)	3 (0.01)
Colon, rectal, and anal cancers								
Overall (*N*, %)	7,325 (2.24)	568 (1.26)***	1,912 (2.62)***	1,394 (2.14)	734 (2.02)**	861 (2.97)***	839 (2.54)***	1,017 (2.23)
Mean age of death (years, SD)	70.13 (0.71)	72.50 (1.20)	73.50 (0.84)	68.72 (0.85)	80.15 (1.24)	70.85 (0.97)	67.23 (0.83)	64.96 (0.81)
Sex								
Female (*N*, %)	3,496 (1.07)	221 (0.49)***	919 (1.26)***	673 (1.03)	429 (1.18)	468 (1.61)***	329 (1.00)	457 (1.00)
Male (*N*, %)	3,829 (1.17)	347 (0.77)***	993 (1.36)***	721 (1.11)	305 (0.84)***	393 (1.36)***	510 (1.54)***	560 (1.23)
Suppressed or sex not stated (*N*, %)	0 (0.00)	0 (0.00)	0 (0.00)	0 (0.00)	0 (0.00)	0 (0.00)	0 (0.00)	0 (0.00)
Age								
< 35 years (*N*, %)	100 (0.03)	15 (0.03)	17 (0.02)	17 (0.03)	—	—	11 (0.03)	31 (0.07)***
35–44 years (*N*, %)	271 (0.08)	42 (0.09)	62 (0.08)	46 (0.07)	10 (0.03)***	14 (0.05)**	31 (0.09)	66 (0.14)***
45–54 years (*N*, %)	835 (0.26)	48 (0.11)***	167 (0.23)	176 (0.27)	34 (0.09)***	100 (0.35)**	138 (0.42)***	172 (0.38)***
55–64 years (*N*, %)	1,424 (0.44)	101 (0.22)***	305 (0.42)	283 (0.43)	100 (0.28)***	174 (0.60)***	208 (0.63)***	253 (0.55)***
65–74 years (*N*, %)	1,723 (0.53)	146 (0.32)***	436 (0.60)*	368 (0.57)	135 (0.37)***	192 (0.66)**	202 (0.61)*	244 (0.53)
75–84 years (*N*, %)	1,581 (0.48)	132 (0.29)***	437 (0.60)***	327 (0.50)	149 (0.41)	231 (0.80)***	148 (0.45)	157 (0.34)***
≥ 85 years (*N*, %)	1,385 (0.42)	82 (0.18)***	487 (0.67)***	176 (0.27)***	302 (0.83)***	144 (0.50)	101 (0.31)**	93 (0.20)***
Suppressed or age not stated (*N*, %)	6 (0.00)	2 (0.00)	1 (0.00)	1 (0.00)	4 (0.01)	5 (0.02)	0 (0.00)	1 (0.00)
Liver and intrahepatic bile duct cancers								
Overall (*N*, %)	6,626 (2.02)	496 (1.10)***	1,585 (2.17)*	1,008 (1.55)***	428 (1.18)***	739 (2.55)***	1,276 (3.86)***	1,094 (2.40)***
Mean age of death (years, SD)	70.77 (0.83)	71.75 (1.31)	72.52 (0.91)	73.02 (1.07)	80.79 (1.67)	75.62 (1.22)	68.94 (0.95)	67.16 (0.95)
Sex								
Female (*N*, %)	2,309 (0.71)	160 (0.35)***	534 (0.73)	428 (0.66)	211 (0.58)**	295 (1.02)***	312 (0.94)***	369 (0.81)*
Male (*N*, %)	4,317 (1.32)	336 (0.74)***	1051 (1.44)*	580 (0.89)***	217 (0.60)***	444 (1.53)**	964 (2.92)***	725 (1.59)***
Suppressed or sex not stated (*N*, %)	0 (0.00)	0 (0.00)	0 (0.00)	0 (0.00)	0 (0.00)	0 (0.00)	0 (0.00)	0 (0.00)
Age								
< 35 years (*N*, %)	50 (0.02)	—	—	—	—	—	—	11 (0.02)
35–44 years (*N*, %)	169 (0.05)	12 (0.03)*	42 (0.06)	19 (0.03)*	—	43 (0.15)***	33 (0.10)***	41 (0.09)**
45–54 years (*N*, %)	493 (0.15)	35 (0.08)***	122 (0.17)	41 (0.06)***	—	96 (0.33)***	105 (0.32)***	137 (0.30)***
55–64 years (*N*, %)	1,375 (0.42)	100 (0.22)***	300 (0.41)	199 (0.31)***	43 (0.12)***	129 (0.45)	325 (0.98)***	285 (0.62)***
65–74 years (*N*, %)	1,896 (0.58)	172 (0.38)***	407 (0.56)	315 (0.48)**	96 (0.26)***	149 (0.51)	411 (1.24)***	330 (0.72)***
75–84 years (*N*, %)	1,695 (0.52)	124 (0.27)***	386 (0.53)	297 (0.46)*	129 (0.36)***	261 (0.90)***	280 (0.85)***	218 (0.48)
≥ 85 years (N, %)	944 (0.29)	47 (0.10)***	319 (0.44)***	129 (0.20)***	149 (0.41)***	122 (0.42)***	110 (0.33)	68 (0.15)***
Suppressed or age not stated (N, %)	4 (0.00)	6 (0.01)	9 (0.01)	8 (0.01)	11 (0.03)	61 (0.21)	12 (0.04)	4 (0.01)
Pancreatic cancers								
Overall (*N*, %)	6,065 (1.85)	559 (1.24)***	1,672 (2.29)***	1,158 (1.78)	752 (2.07)**	757 (2.61)***	506 (1.53)***	661 (1.45)***
Mean age of death (years, SD)	73.09 (0.90)	73.30 (1.32)	74.40 (0.97)	73.24 (1.04)	79.01 (1.21)	73.59 (1.20)	73.71 (1.45)	70.57 (1.25)
Sex								
Female (*N*, %)	3,173 (0.97)	200 (0.44)***	867 (1.19)***	671 (1.03)	456 (1.26)***	443 (1.53)***	229 (0.69)***	307 (0.67)***
Male (*N*, %)	2,892 (0.88)	359 (0.80)	805 (1.10)***	487 (0.75)***	296 (0.81)	314 (1.08)***	277 (0.84)	354 (0.78)*
Suppressed or sex not stated (*N*, %)	0 (0.00)	0 (0.00)	0 (0.00)	0 (0.00)	0 (0.00)	0 (0.00)	0 (0.00)	0 (0.00)
Age								
< 35 years (*N*, %)	14 (0.00)	—	—	—	—	—	—	—
35–44 years (*N*, %)	77 (0.02)	—	16 (0.02)	21 (0.03)	—	—	—	15 (0.03)
45–54 years (*N*, %)	356 (0.11)	33 (0.07)	92 (0.13)	61 (0.09)	23 (0.06)*	45 (0.16)*	49 (0.15)**	53 (0.12)
55–64 years (*N*, %)	981 (0.30)	92 (0.20)***	261 (0.36)*	195 (0.30)	93 (0.26)	127 (0.44)***	83 (0.25)	130 (0.28)
65–74 years (*N*, %)	1,787 (0.55)	193 (0.43)**	492 (0.67)***	354 (0.54)	171 (0.47)	204 (0.70)***	151 (0.46)*	222 (0.49)
75–84 years (*N*, %)	1,819 (0.56)	176 (0.39)***	468 (0.64)**	371 (0.57)	202 (0.56)	273 (0.94)***	162 (0.49)	167 (0.37)***
≥ 85 years (*N*, %)	1,030 (0.31)	54 (0.12)***	340 (0.47)***	156 (0.24)**	257 (0.71)***	101 (0.35)	55 (0.17)***	67 (0.15)***
Suppressed or age not stated (*N*, %)	1 (0.00)	11 (0.02)	3 (0.00)	0 (0.00)	6 (0.02)	7 (0.02)	6 (0.02)	7 (0.02)
Laryngeal cancers								
Overall (*N*, %)	206 (0.06)	38 (0.08)	48 (0.07)	31 (0.05)	16 (0.04)	23 (0.08)	29 (0.09)	21 (0.05)
Mean age of death (years, SD)	—	—	—	—	—	—	—	—
Sex								
Female (*N*, %)	27 (0.01)	—	—	—	—	—	—	—
Male (*N*, %)	179 (0.05)	33 (0.07)	42 (0.06)	30 (0.05)	12 (0.03)	18 (0.06)	28 (0.08)*	16 (0.04)
Suppressed or sex not stated (*N*, %)	0 (0.00)	5 (0.01)	6 (0.01)	1 (0.00)	4 (0.01)	5 (0.02)	1 (0.00)	5 (0.01)
Age								
< 35 years (*N*, %)	—	—	—	—	—	—	—	—
35–44 years (*N*, %)	—	—	—	—	—	—	—	—
45–54 years (*N*, %)	11 (0.00)	—	—	—	—	—	—	—
55–64 years (*N*, %)	33 (0.01)	—	—	—	—	—	—	—
65–74 years (*N*, %)	56 (0.02)	14 (0.03)	12 (0.02)*	16 (0.02)	—	—	—	—
75–84 years (*N*, %)	61 (0.02)	13 (0.03)	16 (0.02)	—	—	—	—	—
≥ 85 years (*N*, %)	45 (0.01)	—	13 (0.02)	—	—	—	—	—
Suppressed or age not stated (*N*, %)	0 (0.00)	11 (0.02)	7 (0.01)	15 (0.02)	16 (0.04)	23 (0.08)	29 (0.09)	21 (0.05)
Tracheal, bronchial, and lung cancers								
Overall (*N*, %)	14,726 (4.50)	996 (2.21)***	4,431 (6.07)***	2,893 (4.44)	1,341 (3.69)***	1,461 (5.04)***	1,954 (5.91)***	1,650 (3.61)***
Mean age of death (years, SD)	73.01 (0.89)	72.96 (1.11)	73.88 (0.88)	73.05 (1.00)	80.59 (1.31)	75.14 (1.13)	70.06 (0.86)	70.12 (0.96)
Sex								
Female (*N*, %)	6,624 (2.02)	341 (0.76)***	2,019 (2.76)***	1,301 (2.00)	818 (2.25)**	731 (2.52)***	685 (2.07)	729 (1.60)***
Male (*N*, %)	8,102 (2.48)	655 (1.45)***	2,412 (3.30)***	1,592 (2.45)	523 (1.44)***	730 (2.52)	1,269 (3.84)***	921 (2.02)***
Suppressed or sex not stated (*N*, %)	0 (0.00)	0 (0.00)	0 (0.00)	0 (0.00)	0 (0.00)	0 (0.00)	0 (0.00)	0 (0.00)
Age								
< 35 years (*N*, %)	40 (0.01)	—	15 (0.02)	—	—	—	—	10 (0.02)
35–44 years (*N*, %)	185 (0.06)	17 (0.04)*	52 (0.07)	21 (0.03)*	—	10 (0.03)	35 (0.11)***	45 (0.10)**
45–54 years (*N*, %)	831 (0.25)	60 (0.13)***	254 (0.35)***	147 (0.23)	12 (0.03)***	49 (0.17)**	177 (0.54)***	132 (0.29)
55–64 years (*N*, %)	2,502 (0.76)	185 (0.41)***	764 (1.05)***	458 (0.70)	97 (0.27)***	180 (0.62)**	475 (1.44)***	343 (0.75)
65–74 years (*N*, %)	4,201 (1.28)	275 (0.61)***	1169 (1.60)***	907 (1.39)*	299 (0.82)***	458 (1.58)**	556 (1.68)***	537 (1.18)
75–84 years (*N*, %)	4,307 (1.32)	326 (0.72)***	1202 (1.65)***	965 (1.48)***	381 (1.05)***	522 (1.80)***	477 (1.44)	434 (0.95)***
≥ 85 years (*N*, %)	2,655 (0.81)	128 (0.28)***	975 (1.33)***	389 (0.60)***	547 (1.51)***	239 (0.82)	229 (0.69)*	148 (0.32)***
Suppressed or age not stated (*N*, %)	5 (0.00)	5 (0.01)	0 (0.00)	6 (0.01)	5 (0.01)	3 (0.01)	5 (0.02)	1 (0.00)

All percentages are calculated using total decedents in each racial/ethnic subgroup as the denominator

^*^p<0.05, **p<0.01, ***p<0.001

— indicates suppressed data values

Further, disaggregated analysis comprised seven Asian American ethnic subgroups. The proportion of cancer-specific deaths within each subroup are as follows: Asian Indians (*n*=45,133; 13.79%), Chinese (*n*=73,051; 22.31%), Filipino (*n*=65,107; 19.89%), Japanese (*n*=36,331; 11.1%), Korean (*n*=28,983; 8.85%), Vietnamese (*n*=33,055; 10.10%), and other Asians (*n*=45,651; 13.95%) (Table 1).

### Asian Indians

Asian Indians comprised 17.49% (*n*=7,892/45,133) of cancer-related deaths, with a mean age of death at 69.10 (SD=0.81) years (Table 1). Among these, females accounted for 44.87% (*n*=3,541/7,892) of deaths. Compared to the aggregated Asian American reference group, Asian Indians exhibited higher mortality rates in lip, oral, and pharyngeal cancers, esophageal cancers, brain and central nervous system cancers, leukemia, and multiple myeloma. Conversely, Asian Indians showed lower mortality rates in total cancers, stomach cancer, colorectal cancer, liver and bile duct cancers, pancreatic cancer, lung cancers, cervical and uterine cancers, non-Hodgkin lymphoma, and certain benign neoplasms (Tables 1 and 2).

**Table 2:** Asian-American skin, reproductive, urinary, hematopoietic, and lymphatic cancer deaths, 2018–2021

Cause of death	Aggregate Asian	Asian Indian	Chinese	Filipino	Japanese	Korean	Vietnamese	Other Asian
Total decedents	327,311 (100.00)	45,133 (100.00)	73,051 (100.00)	65,107 (100.00)	36,331 (100.00)	28,983 (100.00)	33,055 (100.00)	45,651 (100.00)
Skin cancers								
Overall (*N*, %)	219 (0.07)	21 (0.05)	55 (0.08)	38 (0.06)	14 (0.04)***	26 (0.09)	23 (0.07)	42 (0.09)
Mean age of death (years, SD)	70.57 (2.79)	—	—	—	—	—	—	—
Sex								
Female (*N*, %)	111 (0.03)	10 (0.02)	27 (0.04)	23 (0.04)	—	14 (0.05)	10 (0.03)	21 (0.05)
Male (*N*, %)	108 (0.03)	11 (0.02)	28 (0.04)	15 (0.02)	—	12 (0.04)	13 (0.04)	21 (0.05)
Suppressed or sex not stated (N, %)	0 (0.00)	0 (0.00)	0 (0.00)	0 (0.00)	14 (0.04)	0 (0.00)	0 (0.00)	0 (0.00)
Age								
< 35 years (*N*, %)	10 (0.00)	—	—	—	—	—	—	—
35–44 years (*N*, %)	10 (0.00)	—	—	—	—	—	—	—
45–54 years (*N*, %)	21 (0.01)	—	—	—	—	—	—	—
55–64 years (*N*, %)	45 (0.01)	—	12 (0.02)	—	—	—	—	11 (0.02)
65–74 years (N, %)	51 (0.02)	—	14 (0.02)	—	—	—	—	10 (0.02)
75–84 years (*N*, %)	51 (0.02)	—	12 (0.02)	10 (0.02)	—	—	—	—
≥ 85 years (*N*, %)	31 (0.01)	—	—	—	—	—	—	—
Suppressed or age not stated (*N*, %)	0 (0.00)	21 (0.05)	17 (0.02)	28 (0.04)	14 (0.04)	26 (0.09)	23 (0.07)	21 (0.05)
Breast cancers								
Overall (*N*, %)	5,273 (1.61)	707 (1.57)	1,170 (1.60)	1541 (2.37)***	488 (1.34)***	375 (1.29)***	341 (1.03)***	651 (1.43)
Mean age of death (years, SD)	65.53 (0.62)	64.94 (0.90)	66.93 (0.67)	65.05 (0.75)	79.62 (1.53)	66.10 (1.74)	61.70 (2.11)	60.41 (0.98)
Sex								
Female (*N*, %)	5,241 (1.60)	699 (1.55)	1161 (1.59)	1534 (2.36)***	487 (1.34)***	373 (1.29)***	340 (1.03)***	647 (1.42)
Male (*N*, %)	32 (0.01)	—	—	—	—	—	—	—
Suppressed or sex not stated (*N*, %)	0 (0.00)	8 (0.02)	9 (0.01)	7 (0.01)	1 (0.00)	2 (0.01)	1 (0.00)	4 (0.01)
Age								
< 35 years (*N*, %)	58 (0.02)	13 (0.03)	16 (0.02)	—	—	—	—	14 (0.03)
35–44 years (*N*, %)	374 (0.11)	71 (0.16)*	76 (0.10)	105 (0.16)**	13 (0.04)***	27 (0.09)	30 (0.09)	52 (0.11)
45–54 years (*N*, %)	896 (0.27)	117 (0.26)	208 (0.28)	253 (0.39)**	27 (0.07)***	68 (0.23)	85 (0.26)	138 (0.30)
55–64 years (*N*, %)	1,235 (0.38)	147 (0.33)	269 (0.37)	376 (0.58)***	80 (0.22)***	92 (0.32)	96 (0.29)*	175 (0.38)
65–74 years (*N*, %)	1,207 (0.37)	174 (0.39)	241 (0.33)	397 (0.61)***	102 (0.28)**	80 (0.28)*	59 (0.18)***	154 (0.34)
75–84 years (*N*, %)	832 (0.25)	122 (0.27)	177 (0.24)	265 (0.41)***	84 (0.23)	72 (0.25)	45 (0.14)***	67 (0.15)***
≥ 85 years (*N*, %)	669 (0.20)	63 (0.14)**	182 (0.25)*	136 (0.21)	181 (0.50)***	34 (0.12)**	22 (0.07)***	51 (0.11)***
Suppressed or age not stated (*N*, %)	2 (0.00)	0 (0.00)	1 (0.00)	9 (0.01)	1 (0.00)	2 (0.01)	4 (0.01)	0 (0.00)
Cervical and uterine cancers								
Overall (*N*, %)	2,256 (0.69)	258 (0.57)**	505 (0.69)	614 (0.94)***	183 (0.50)***	166 (0.57)*	226 (0.68)	304 (0.67)
Mean age of death (years, SD)	65.63 (3.29)	70.72 (5.71)	65.73 (3.73)	62.08 (4.02)	73.18 (5.89)	68.65 (6.03)	60.10 (4.76)	60.65 (4.75)
Sex								
Female (*N*, %)	2,256 (0.69)	258 (0.57)**	505 (0.69)	614 (0.94)***	183 (0.50)***	166 (0.57)*	226 (0.68)	304 (0.67)
Male (*N*, %)	0 (0.00)	0 (0.00)	0 (0.00)	0 (0.00)	0 (0.00)	0 (0.00)	0 (0.00)	0 (0.00)
Suppressed or sex not stated (*N*, %)	0 (0.00)	0 (0.00)	0 (0.00)	0 (0.00)	0 (0.00)	0 (0.00)	0 (0.00)	0 (0.00)
Age								
< 35 years (*N*, %)	28 (0.01)	—	—	—	—	—	—	—
35–44 years (*N*, %)	111 (0.03)	—	11 (0.02)*	30 (0.05)	—	—	12 (0.04)	28 (0.06)**
45–54 years (*N*, %)	361 (0.11)	17 (0.04)***	90 (0.12)	89 (0.14)	—	31 (0.11)	51 (0.15)*	56 (0.12)
55–64 years (*N*, %)	557 (0.17)	35 (0.08)***	143 (0.20)	163 (0.25)**	43 (0.12)*	41 (0.14)	54 (0.16)	69 (0.15)
65–74 years (*N*, %)	601 (0.18)	86 (0.19)	126 (0.17)	175 (0.27)***	38 (0.10)***	44 (0.15)	55 (0.17)	68 (0.15)
75–84 years (*N*, %)	388 (0.12)	57 (0.13)	67 (0.09)	101 (0.16)*	34 (0.09)	21 (0.07)**	36 (0.11)	54 (0.12)
≥ 85 years (*N*, %)	209 (0.06)	21 (0.05)	56 (0.08)	48 (0.07)	30 (0.08)	—	—	14 (0.03)**
Suppressed or age not stated (*N*, %)	1 (0.00)	42 (0.09)	12 (0.02)	8 (0.01)	38 (0.10)	29 (0.10)	18 (0.05)	15 (0.03)
Ovarian cancers								
Overall (*N*, %)	1,988 (0.61)	309 (0.68)	455 (0.62)	446 (0.69)*	149 (0.41)***	204 (0.70)*	171 (0.52)*	254 (0.56)
Mean age of death (years, SD)	65.99 (0.77)	64.58 (2.25)	62.13 (1.37)	64.70 (1.45)	73.31 (4.47)	66.97 (2.81)	61.86 (3.13)	63.78 (3.00)
Sex								
Female (*N*, %)	1,988 (0.61)	309 (0.68)	455 (0.62)	446 (0.69)*	149 (0.41)***	204 (0.70)*	171 (0.52)*	254 (0.56)
Male (*N*, %)	0 (0.00)	0 (0.00)	0 (0.00)	0 (0.00)	0 (0.00)	0 (0.00)	0 (0.00)	0 (0.00)
Suppressed or sex not stated (*N*, %)								
Age	(0.61)	(0.68)	(0.62)	(0.69)	(0.41)	(0.70)	(0.52)	(0.56)
< 35 years (*N*, %)	26 (0.01)	—	—	—	—	—	—	—
35–44 years (*N*, %)	76 (0.02)	13 (0.03)	18 (0.02)	17 (0.03)	—	—	—	14 (0.03)
45–54 years (*N*, %)	333 (0.10)	51 (0.11)	91 (0.12)	59 (0.09)	13 (0.04)***	34 (0.12)	35 (0.11)	50 (0.11)
55–64 years (*N*, %)	515 (0.16)	82 (0.18)	102 (0.14)	124 (0.19)	30 (0.08)***	50 (0.17)	51 (0.15)	76 (0.17)
65–74 years (*N*, %)	527 (0.16)	90 (0.20)	101 (0.14)	146 (0.22)***	32 (0.09)***	48 (0.17)	49 (0.15)	61 (0.13)
75–84 years (*N*, %)	329 (0.10)	50 (0.11)	80 (0.11)	70 (0.11)	36 (0.10)	45 (0.16)**	20 (0.06)*	28 (0.06)*
≥ 85 years (*N*, %)	176 (0.05)	19 (0.04)	56 (0.08)*	23 (0.04)	35 (0.10)**	17 (0.06)	10 (0.03)	16 (0.04)
Suppressed or age not stated (*N*, %)	6 (0.00)	4 (0.01)	7 (0.01)	7 (0.01)	3 (0.01)	10 (0.03)	6 (0.02)	9 (0.02)
Prostate cancers								
Overall (*N*, %)	2,589 (0.79)	383 (0.85)	583 (0.80)	667 (1.02)***	308 (0.85)	181 (0.62)**	210 (0.64)**	257 (0.56)***
Mean age of death (years, SD)	80.65 (1.16)	80.36 (1.90)	84.48 (1.54)	80.47 (1.40)	85.32 (7.76)	80.73 (5.07)	79.36 (4.91)	75.00 (2.74)
Sex								
Female (*N*, %)	0 (0.00)	0 (0.00)	0 (0.00)	0 (0.00)	0 (0.00)	0 (0.00)	0 (0.00)	0 (0.00)
Male (*N*, %)	2589 (0.79)	383 (0.85)	583 (0.80)	667 (1.02)***	308 (0.85)	181 (0.62)**	210 (0.64)**	257 (0.56)***
Suppressed or sex not stated (*N*, %)	0 (0.00)	0 (0.00)	0 (0.00)	0 (0.00)	0 (0.00)	0 (0.00)	0 (0.00)	0 (0.00)
Age								
< 35 years (*N*, %)	—	—	—	—	—	—	—	—
35–44 years (*N*, %)	—	—	—	—	—	—	—	—
45–54 years (*N*, %)	28 (0.01)	—	—	13 (0.02)*	—	—	—	—
55–64 years (*N*, %)	162 (0.05)	29 (0.06)	26 (0.04)	49 (0.08)*	11 (0.03)	—	16 (0.05)	24 (0.05)
65–74 years (*N*, %)	544 (0.17)	102 (0.23)**	89 (0.12)**	148 (0.23)***	42 (0.12)*	37 (0.13)	48 (0.15)	78 (0.17)
75–84 years (*N*, %)	849 (0.26)	130 (0.29)	186 (0.25)	244 (0.37)***	68 (0.19)*	66 (0.23)	75 (0.23)	80 (0.18)***
≥ 85 years (*N*, %)	1,005 (0.31)	119 (0.26)	279 (0.38)**	213 (0.33)	187 (0.51)***	70 (0.24)	71 (0.21)**	66 (0.14)***
Suppressed or age not stated (*N*, %)	1 (0.00)	3 (0.01)	3 (0.00)	0 (0.00)	0 (0.00)	8 (0.03)	0 (0.00)	9 (0.02)
Kidney cancers								
Overall (*N*, %)	1,203 (0.37)	137 (0.30)*	276 (0.38)	283 (0.43)*	137 (0.38)	124 (0.43)	105 (0.32)	141 (0.31)
Mean age of death (years, SD)	72.80 (0.92)	—	84.28 (3.36)	70.57 (2.30)	—	—	—	—
Sex								
Female (*N*, %)	426 (0.13)	36 (0.08)**	99 (0.14)	108 (0.17)*	53 (0.15)	51 (0.18)*	32 (0.10)	47 (0.10)
Male (*N*, %)	777 (0.24)	101 (0.22)	177 (0.24)	175 (0.27)	84 (0.23)	73 (0.25)	73 (0.22)	94 (0.21)
Suppressed or sex not stated (*N*, %)	0 (0.00)	0 (0.00)	0 (0.00)	0 (0.00)	0 (0.00)	0 (0.00)	0 (0.00)	0 (0.00)
Age								
< 35 years (*N*, %)	—	—	—	—	—	—	—	—
35–44 years (*N*, %)	19 (0.01)	—	—	—	—	—	—	—
45–54 years (*N*, %)	93 (0.03)	15 (0.03)	24 (0.03)	14 (0.02)	—	—	13 (0.04)	15 (0.03)
55–64 years (*N*, %)	219 (0.07)	25 (0.06)	41 (0.06)	62 (0.10)*	21 (0.06)	24 (0.08)	22 (0.07)	24 (0.05)
65–74 years (*N*, %)	332 (0.10)	48 (0.11)	68 (0.09)	85 (0.13)*	35 (0.10)	27 (0.09)	23 (0.07)	46 (0.10)
75–84 years (*N*, %)	312 (0.10)	34 (0.08)	54 (0.07)	82 (0.13)*	43 (0.12)	41 (0.14)*	27 (0.08)	31 (0.07)
≥ 85 years (*N*, %)	214 (0.07)	13 (0.03)**	85 (0.12)	34 (0.05)	35 (0.10)*	18 (0.06)	16 (0.05)	13 (0.03)**
Suppressed or age not stated (*N*, %)	14 (0.00)	2 (0.00)	4 (0.01)	6 (0.01)	3 (0.01)	14 (0.05)	4 (0.01)	12 (0.03)
Bladder cancers								
Overall (*N*, %)	1,230 (0.38)	154 (0.34)	315 (0.43)*	185 (0.28)***	171 (0.47)**	167 (0.58)***	98 (0.30)*	140 (0.31)*
Mean age of death (years, SD)	80.10 (1.11)	79.75 (8.18)	83.34 (6.69)	77.21 (6.49)	82.52 (6.67)	80.86 (8.68)	77.71 (8.17)	73.98 (9.45)
Sex								
Female (*N*, %)	379 (0.12)	29 (0.06)**	117 (0.16)**	51 (0.08)*	71 (0.20)***	45 (0.16)	27 (0.08)	39 (0.09)
Male (*N*, %)	851 (0.26)	125 (0.28)	198 (0.27)	134 (0.21)*	100 (0.28)	122 (0.42)***	71 (0.21)	101 (0.22)
Suppressed or sex not stated (*N*, %)	0 (0.00)	0 (0.00)	0 (0.00)	0 (0.00)	0 (0.00)	0 (0.00)	0 (0.00)	0 (0.00)
Age								
< 35 years (*N*, %)	—	—	—	—	—	—	—	—
35–44 years (*N*, %)	12 (0.00)	—	—	—	—	—	—	—
45–54 years (*N*, %)	31 (0.01)	—	—	—	—	—	—	—
55–64 years (*N*, %)	112 (0.03)	16 (0.04)	19 (0.03)	23 (0.04)	12 (0.03)	13 (0.04)	13 (0.04)	16 (0.04)
65–74 years (*N*, %)	260 (0.08)	27 (0.06)	48 (0.07)	46 (0.07)	28 (0.08)	33 (0.11)	23 (0.07)	55 (0.12)**
75–84 years (*N*, %)	386 (0.12)	65 (0.14)	88 (0.12)	63 (0.10)	38 (0.10)	64 (0.22)***	32 (0.10)	36 (0.08)*
≥ 85 years (*N*, %)	428 (0.13)	41 (0.09)*	153 (0.21)***	43 (0.07)***	91 (0.25)***	53 (0.18)*	25 (0.08)**	22 (0.05)***
Suppressed or age not stated (*N*, %)	1 (0.00)	5 (0.01)	7 (0.01)	10 (0.02)	2 (0.01)	4 (0.01)	5 (0.02)	11 (0.02)
Brain, meningeal, or other central nervous system cancers								
Overall (*N*, %)	1,873 (0.57)	407 (0.90)***	416 (0.57)	358 (0.55)	112 (0.31)***	126 (0.43)**	182 (0.55)	272 (0.60)
Mean age of death (years, SD)	65.00 (0.70)	68.84 (1.90)	67.83 (2.09)	68.25 (2.51)	71.92 (7.42)	66.80 (6.89)	62.87 (4.97)	57.42 (3.36)
Sex								
Female (*N*, %)	819 (0.25)	152 (0.34)***	178 (0.24)	173 (0.27)	60 (0.17)**	64 (0.22)	88 (0.27)	104 (0.23)
Male (*N*, %)	1,054 (0.32)	255 (0.56)***	238 (0.33)	185 (0.28)	52 (0.14)***	62 (0.21)**	94 (0.28)	168 (0.37)
Suppressed or sex not stated (*N*, %)	0 (0.00)	0 (0.00)	0 (0.00)	0 (0.00)	0 (0.00)	0 (0.00)	0 (0.00)	0 (0.00)
Age								
< 35 years (*N*, %)	189 (0.06)	27 (0.06)	36 (0.05)	15 (0.02)	—	—	—	37 (0.08)
35–44 years (*N*, %)	125 (0.04)	26 (0.06)	18 (0.02)	30 (0.05)	—	—	15 (0.05)	27 (0.06)
45–54 years (*N*, %)	242 (0.07)	57 (0.13)***	49 (0.07)	43 (0.07)	—	14 (0.05)	30 (0.09)	40 (0.09)
55–64 years (*N*, %)	391 (0.12)	79 (0.18)**	88 (0.12)	69 (0.11)	24 (0.07)**	34 (0.12)	38 (0.11)	59 (0.13)
65–74 years (*N*, %)	427 (0.13)	105 (0.23)***	98 (0.13)	89 (0.14)	19 (0.05)***	21 (0.07)**	38 (0.11)	57 (0.12)
75–84 years (*N*, %)	312 (0.10)	75 (0.17)***	61 (0.08)	66 (0.10)	22 (0.06)**	28 (0.10)	29 (0.09)	31 (0.07)
≥ 85 years (*N*, %)	184 (0.06)	25 (0.06)	64 (0.09)**	33 (0.05)	28 (0.08)	11 (0.04)	12 (0.04)	11 (0.02)**
Suppressed or age not stated (*N*, %)	3 (0.00)	13 (0.03)	2 (0.00)	13 (0.02)	19 (0.05)	18 (0.06)	20 (0.06)	10 (0.02)
Hodgkin disease								
Overall (*N*, %)	74 (0.02)	16 (0.04)	12 (0.02)	14 (0.02)	—	—	—	11 (0.02)
Mean age of death (years, SD)	—	—	—	—	—	—	—	—
Sex								
Female (*N*, %)	35 (0.01)	12 (0.03)**	—	—	—	—	—	—
Male (*N*, %)	39 (0.01)	0 (0.00)*	—	—	—	—	—	—
Suppressed or sex not stated (*N*, %)	0 (0.00)	4 (0.01)	12 (0.02)	14 (0.02)	—	—	—	11 (0.02)
Age								
< 35 years (*N*, %)	—	—	—	—	—	—	—	—
35–44 years (*N*, %)	—	—	—	—	—	—	—	—
45–54 years (*N*, %)	—	—	—	—	—	—	—	—
55–64 years (*N*, %)	13 (0.00)	—	—	—	—	—	—	—
65–74 years (*N*, %)	13 (0.00)	—	—	—	—	—	—	—
75–84 years (*N*, %)	18 (0.01)	—	—	—	—	—	—	—
≥ 85 years (*N*, %)	19 (0.01)	—	—	—	—	—	—	—
Suppressed or age not stated (*N*, %)	11 (0.00)	16 (0.04)	12 (0.02)	14 (0.02)	—	—	—	11 (0.02)
Non-Hodgkin lymphoma								
Overall (*N*, %)	2,765 (0.84)	338 (0.75)*	661 (0.90)	624 (0.96)**	302 (0.83)	207 (0.71)*	305 (0.92)	328 (0.72)**
Mean age of death (years, SD)	74.56 (0.95)	72.80 (1.95)	78.13 (1.21)	74.17 (1.22)	81.53 (7.02)	73.38 (6.01)	74.89 (2.15)	71.50 (2.19)
Sex								
Female (*N*, %)	1,218 (0.37)	119 (0.26)	282 (0.39)	298 (0.46)**	161 (0.44)*	97 (0.33)	107 (0.32)	154 (0.34)
Male (*N*, %)	1,547 (0.47)	219 (0.49)	379 (0.52)	326 (0.50)	141 (0.39)*	110 (0.38)*	198 (0.60)**	174 (0.38)**
Suppressed or sex not stated (*N*, %)	0 (0.00)	0 (0.00)	0 (0.00)	0 (0.00)	0 (0.00)	0 (0.00)	0 (0.00)	0 (0.00)
Age								
< 35 years (*N*, %)	36 (0.01)	—	—	—	—	—	—	—
35–44 years (*N*, %)	56 (0.02)	—	15 (0.02)	—	—	—	10 (0.03)	11 (0.02)
45–54 years (*N*, %)	126 (0.04)	22 (0.05)	23 (0.03)	26 (0.04)	—	—	23 (0.07)*	21 (0.05)
55–64 years (*N*, %)	406 (0.12)	65 (0.14)	77 (0.11)	100 (0.15)	24 (0.07)**	39 (0.13)	43 (0.13)	58 (0.13)
65–74 years (*N*, %)	731 (0.22)	79 (0.18)*	162 (0.22)	184 (0.28)**	53 (0.15)**	50 (0.17)	94 (0.28)*	109 (0.24)
75–84 years (*N*, %)	832 (0.25)	118 (0.26)	192 (0.26)	198 (0.30)*	87 (0.24)	63 (0.22)	97 (0.29)	77 (0.17)***
≥ 85 years (*N*, %)	574 (0.18)	42 (0.09)***	188 (0.26)***	101 (0.16)	132 (0.36)***	37 (0.13)	32 (0.10)**	42 (0.09)***
Suppressed or age not stated (*N*, %)	4 (0.00)	12 (0.03)	4 (0.01)	15 (0.02)	6 (0.02)	18 (0.06)	6 (0.02)	10 (0.02)
Leukemia								
Overall (*N*, %)	2,518 (0.77)	392 (0.87)*	645 (0.88)**	563 (0.86)*	185 (0.51)***	168 (0.58)***	260 (0.79)	305 (0.67)*
Mean age of death (years, SD)	71.97 (0.90)	75.93 (2.63)	71.99 (4.38)	75.62 (1.46)	80.31 (7.17)	80.56 (5.65)	72.41 (5.61)	72.73 (3.22)
Sex								
Female (*N*, %)	1,100 (0.34)	156 (0.35)	278 (0.38)	264 (0.41)**	91 (0.25)**	79 (0.27)	94 (0.28)	138 (0.30)
Male (*N*, %)	1,418 (0.43)	236 (0.52)**	367 (0.50)*	299 (0.46)	94 (0.26)***	89 (0.31)**	166 (0.50)	167 (0.37)*
Suppressed or sex not stated (*N*, %)	0 (0.00)	0 (0.00)	0 (0.00)	0 (0.00)	0 (0.00)	0 (0.00)	0 (0.00)	0 (0.00)
Age								
< 35 years (*N*, %)	175 (0.05)	26 (0.06)	28 (0.04)	10 (0.02)***	—	—	—	34 (0.07)
35–44 years (*N*, %)	91 (0.03)	20 (0.04)	25 (0.03)	15 (0.02)	—	—	—	12 (0.03)
45–54 years (*N*, %)	167 (0.05)	36 (0.08)*	38 (0.05)	36 (0.06)	—	—	16 (0.05)	18 (0.04)
55–64 years (*N*, %)	357 (0.11)	62 (0.14)	81 (0.11)	82 (0.13)	13 (0.04)***	18 (0.06)*	44 (0.13)	57 (0.12)
65–74 years (*N*, %)	589 (0.18)	85 (0.19)	157 (0.21)	139 (0.21)	44 (0.12)*	34 (0.12)*	61 (0.18)	69 (0.15)
75–84 years (*N*, %)	718 (0.22)	86 (0.19)	187 (0.26)	181 (0.28)**	51 (0.14)**	65 (0.22)	78 (0.24)	70 (0.15)**
≥ 85 years (*N*, %)	415 (0.13)	57 (0.13)	113 (0.15)	87 (0.13)	64 (0.18)*	24 (0.08)	38 (0.11)	32 (0.07)**
Suppressed or age not stated (*N*, %)	6 (0.00)	20 (0.04)	16 (0.02)	13 (0.02)	13 (0.04)	27 (0.09)	23 (0.07)	13 (0.03)
Multiple myeloma and immunoproliferative neoplasms								
Overall (*N*, %)	1,167 (0.36)	197 (0.44)**	256 (0.35)	300 (0.46)***	84 (0.23)***	92 (0.32)	109 (0.33)	129 (0.28)*
Mean age of death (years, SD)	75.33 (1.09)	75.34 (6.98)	76.54 (5.76)	73.94 (6.30)	87.19 (12.71)	79.84 (9.85)	69.05 (6.13)	73.61 (4.55)
Sex								
Female (*N*, %)	536 (0.16)	73 (0.16)	105 (0.14)	156 (0.24)***	43 (0.12)*	50 (0.17)	51 (0.15)	58 (0.13)
Male (*N*, %)	631 (0.19)	124 (0.27)***	151 (0.21)	144 (0.22)	41 (0.11)***	42 (0.14)	58 (0.18)	71 (0.16)
Suppressed or sex not stated (*N*, %)	0 (0.00)	0 (0.00)	0 (0.00)	0 (0.00)	0 (0.00)	0 (0.00)	0 (0.00)	0 (0.00)
Age								
< 35 years (*N*, %)	—	—	—	—	—	—	—	—
35–44 years (*N*, %)	—	—	—	—	—	—	—	—
45–54 years (*N*, %)	39 (0.01)	—	—	12 (0.02)	—	—	—	—
55–64 years (*N*, %)	168 (0.05)	27 (0.06)	34 (0.05)	42 (0.06)	10 (0.03)	12 (0.04)	19 (0.06)	24 (0.05)
65–74 years (*N*, %)	320 (0.10)	63 (0.14)*	66 (0.09)	98 (0.15)***	11 (0.03)***	21 (0.07)	33 (0.10)	28 (0.06)*
75–84 years (*N*, %)	390 (0.12)	71 (0.16)*	80 (0.11)	102 (0.16)*	18 (0.05)***	39 (0.13)	33 (0.10)	47 (0.10)
≥ 85 years (*N*, %)	241 (0.07)	30 (0.07)	68 (0.09)	44 (0.07)	44 (0.12)**	19 (0.07)	17 (0.05)	19 (0.04)*
Suppressed or age not stated (*N*, %)	9 (0.00)	6 (0.01)	8 (0.01)	2 (0.00)	1 (0.00)	1 (0.00)	7 (0.02)	11 (0.02)
Other and unspecified cancers								
Overall (*N*, %)	8,797 (2.69)	1,178 (2.61)	2,155 (2.95)***	1,726 (2.65)	868 (2.39)***	818 (2.82)	921 (2.79)	1,131 (2.48)**
Mean age of death (years, SD)	67.83 (0.88)	67.32 (3.83)	71.49 (3.99)	70.88 (4.24)	78.80 (5.73)	72.78 (4.54)	69.42 (3.79)	63.96 (3.23)
Sex								
Female (*N*, %)	4,459 (1.36)	533 (1.18)**	1,058 (1.45)	989 (1.52)**	514 (1.41)	441 (1.52)*	381 (1.15)**	543 (1.19)**
Male (*N*, %)	4,338 (1.33)	645 (1.43)	1,097 (1.50)***	737 (1.13)***	354 (0.97)***	377 (1.30)	540 (1.63)***	588 (1.29)
Suppressed or sex not stated (*N*, %)	0 (0.00)	0 (0.00)	0 (0.00)	0 (0.00)	0 (0.00)	0 (0.00)	0 (0.00)	0 (0.00)
Age								
< 35 years (*N*, %)	293 (0.09)	57 (0.13)	69 (0.09)	28 (0.04)***	—	—	19 (0.06)	70 (0.15)***
35–44 years (*N*, %)	311 (0.10)	51 (0.11)	69 (0.09)	59 (0.09)	—	24 (0.08)	27 (0.08)	74 (0.16)***
45–54 years (*N*, %)	705 (0.22)	126 (0.28)**	144 (0.20)	131 (0.20)	25 (0.07)***	52 (0.18)**	99 (0.30)	128 (0.28)**
55–64 years (*N*, %)	1,489 (0.45)	224 (0.50)	340 (0.47)	288 (0.44)	103 (0.28)***	143 (0.49)	166 (0.50)	225 (0.49)
65–74 years (*N*, %)	2,206 (0.67)	315 (0.70)	517 (0.71)	489 (0.75)*	174 (0.48)***	174 (0.60)	245 (0.74)	292 (0.64)
75–84 years (*N*, %)	2,200 (0.67)	284 (0.63)	515 (0.70)	476 (0.73)	209 (0.58)*	257 (0.89)***	230 (0.70)	229 (0.50)***
≥ 85 years (*N*, %)	1,589 (0.49)	117 (0.26)***	497 (0.68)***	250 (0.38)***	343 (0.94)***	151 (0.52)	126 (0.38)**	105 (0.23)***
Suppressed or age not stated (*N*, %)	4 (0.00)	4 (0.01)	4 (0.01)	5 (0.01)	14 (0.04)	17 (0.06)	9 (0.03)	8 (0.02)
Neoplasms in situ, benign, or of uncertain or unknown behavior								
Overall (*N*, %)	1,861 (0.57)	209 (0.46)**	436 (0.60)	417 (0.64)*	204 (0.56)	168 (0.58)	197 (0.60)	230 (0.50)
Mean age of death (years, SD)	78.28 (1.02)	77.06 (6.36)	84.88 (1.83)	79.21 (1.68)	87.27 (3.82)	79.02 (6.65)	76.67 (5.90)	84.88 (1.83)
Sex								
Female (*N*, %)	894 (0.27)	88 (0.19)**	198 (0.27)	219 (0.34)**	109 (0.30)	78 (0.27)	97 (0.29)	105 (0.23)
Male (*N*, %)	967 (0.30)	121 (0.27)	238 (0.33)	198 (0.30)	95 (0.26)	90 (0.31)	100 (0.30)	125 (0.27)
Suppressed or sex not stated (*N*, %)	0 (0.00)	0 (0.00)	0 (0.00)	0 (0.00)	0 (0.00)	0 (0.00)	0 (0.00)	0 (0.00)
Age								
< 35 years (*N*, %)	60 (0.02)	—	—	—	—	—	—	—
35–44 years (*N*, %)	54 (0.02)	—	—	13 (0.02)	—	—	—	10 (0.02)
45–54 years (*N*, %)	80 (0.02)	11 (0.02)	14 (0.02)	15 (0.02)	—	15 (0.05)*	10 (0.03)	12 (0.03)
55–64 years (*N*, %)	177 (0.05)	22 (0.05)	30 (0.04)	40 (0.06)	10 (0.03)*	18 (0.06)*	29 (0.09)*	28 (0.06)
65–74 years (*N*, %)	353 (0.11)	43 (0.10)	71 (0.10)	100 (0.15)**	23 (0.06)*	30 (0.10)	36 (0.11)	50 (0.11)
75–84 years (*N*, %)	539 (0.16)	67 (0.15)	120 (0.16)	136 (0.21)*	43 (0.12)*	45 (0.16)	58 (0.18)	70 (0.15)
≥ 85 years (*N*, %)	589 (0.18)	45 (0.10)***	178 (0.24)***	106 (0.16)	123 (0.34)***	50 (0.17)	47 (0.14)	40 (0.09)***
Suppressed or age not stated (*N*, %)	9 (0.00)	21 (0.05)	23 (0.03)	7 (0.01)	5 (0.01)	10 (0.03)	17 (0.05)	20 (0.04)

All percentages are calculated using total decedents in each racial/ethnic subgroup as the denominator

^*^p<0.05, **p<0.01, ***p<0.001

— indicates suppressed data values

Gender-stratified analysis revealed that Asian Indian females were more prone to Hodgkin’s lymphoma and unspecified or other cancers, but less likely to die from kidney and bladder cancers compared to aggregated Asian American females. Asian Indian females also had a higher mortality rate from cervical and uterine cancers (Tables 1 and 2). Age-stratified analysis indicated higher mortality rates in lip, oral, and pharyngeal cancers (ages 55–84), prostate cancer (ages 65–75), and brain and central nervous system cancers (ages 45–84). Conversely, Asian Indians were less likely to die from liver and bile duct cancers, lung cancers, total and colorectal cancers, stomach and pancreatic cancers, cervical and uterine cancers, kidney and bladder cancers, and non-Hodgkin lymphoma in specific age groups compared to aggregated Asian Americans (Tables 1 and 2).

### Chinese

Chinese individuals accounted for 25.99% (*n*=18,987/73,051) of cancer deaths, with a mean age of death at 72.33 (SD=0.79) years (Table 1). About half (48.97%) of them were female. Compared to the aggregated Asian American reference group, Chinese individuals had higher mortality rates in various cancers including total cancers, lip, oral, and pharyngeal cancers, stomach cancer, colorectal cancer, liver and bile duct cancers, pancreatic cancer, lung cancers, bladder cancer, leukemia, and unspecified or other cancers (Tables 1–2).

Gender-stratified analysis showed no significant differences in mortality rates between Chinese females and aggregated Asian American females. Similarly, Chinese males did not exhibit significant differences in prostate cancer mortality compared to aggregated Asian American males (Tables 1 and 2). Age-stratified analysis suggested higher mortality rates in lip, oral, and pharyngeal cancers (ages 35–54 and 65–84), stomach and pancreatic cancers (above age 65), colorectal cancer (above age 65), and various other neoplasms among Chinese individuals in specific age groups compared to aggregated Asian Americans (Tables 1 and 2).

### Filipino

Filipino individuals represented 22.38% (*n*=14,571/65,107) of cancer deaths, with a mean age of death at 70.57 (SD=0.83) years (Table 1). Females constituted a slight majority (55.15%) of the sample. Compared to the aggregated Asian American reference group, Filipinos showed higher mortality rates in breast cancer, cervical and uterine cancers, ovarian cancer, prostate cancer, kidney cancer, non-Hodgkin lymphoma, leukemia, multiple myeloma, and certain benign neoplasms. Conversely, they exhibited lower mortality rates in lip, oral, and pharyngeal cancers, esophageal cancer, stomach cancer, liver and bile duct cancers, and bladder cancer (Tables 1 and 2).

Gender-stratified analysis indicated higher mortality rates in total cancers, breast cancers, cervical and uterine cancers, ovarian cancers, and unspecified or other cancers among Filipina females compared to aggregated Asian American females. Filipino males exhibited a higher mortality rate from prostate cancer (Tables 1–2). Relative to aggregated Asian Americans, Filipinos exhibited higher mortality rates from certain cancers, including prostate cancer (ages 45–84), breast, cervical, uterine, and kidney cancers (ages 55–84), and ovarian cancer and other unspecified cancers (ages 65–74). Conversely, they showed lower mortality rates from liver and intrahepatic bile duct cancers (above age 35), breast cancer (ages 35–84), and various cancers like tracheal, bronchial, and lung cancers (ages 35–44 or above 85), among others, compared to the aggregated Asian American population (Tables 1 and 2).

### Japanese

Japanese individuals, comprising 18.90% (*n*=6,868/36,331) of the sample, demonstrated distinct cancer mortality patterns (Table 1). Compared to Asian Americans, Japanese individuals had higher rates of esophageal, pancreatic, and bladder cancers but lower rates of lip, oral, and pharyngeal, stomach, colorectal, liver, tracheal, bronchial, and lung, skin, breast, cervical and uterine, ovarian, brain, leukemia, and multiple myeloma (Tables 1 and 2).

Gender-stratified analysis showed Japanese females had higher mortality from tracheal, bronchial, and lung cancers and non-Hodgkin lymphoma but lower mortality from breast, cervical and uterine, and ovarian cancers compared to Asian American females. Japanese males had lower mortality from tracheal, bronchial, and lung cancers and non-Hodgkin lymphoma compared to Asian American males (Tables 1 and 2). Further, age-stratified analysis revealed higher mortality among Japanese individuals aged above 85 for esophageal, kidney, and bladder cancers. Conversely, Japanese had higher mortality from colorectal and cervical and uterine cancers at younger ages compared to Asian Americans (Tables 1 and 2).

### Korean

In total, 25.29% (*n*=7,330/28,983) Korean individuals died of cancer, with a mean age of death of 72.34 (SD=0.88) years, and approximately half (*n*=3,866/7,330; 52.74%) identified as female (Table 1). Koreans had higher mortality rates for various cancers including stomach, colorectal, pancreatic, liver, tracheal, bronchial, and lung, and bladder cancers but lower rates in cancer sites such as the lip, oral, and pharyngeal, breast, cervical and uterine, prostate, brain, non-Hodgkin lymphoma, and leukemia (Tables 1 and 2).

Gender-stratified analysis showed Korean females had higher mortality from kidney cancer and unspecified or other cancers but lower mortality from breast and cervical and uterine cancers compared to Asian American females. Korean males had lower mortality from prostate cancer compared to Asian American males (Tables 1 and 2).

Age-stratified analysis indicated higher mortality among Koreans for liver and intrahepatic bile duct cancers (ages 35–54 or above 75), stomach cancer (above age 45), colorectal and pancreatic cancers (ages 45–84), tracheal, bronchial, and lung cancers (ages 65–84), ovarian, kidney, and unspecified or other cancers (ages 75–84), and bladder cancer (above age 75). Conversely, Koreans had lower mortality from several cancers at certain age intervals compared to Asian Americans (Tables 1 and 2).

### Vietnamese

Overall, 24.98% (*n*=8,258/33,055) Vietnamese individuals died of cancer, with a mean age of death of 68.59 (SD=0.76) years, and females accounted for a minority (39.83%) (Table 1). Vietnamese individuals had higher mortality rates for total cancers, tracheal, bronchial, and lung, colorectal, and liver cancers but lower rates of pancreatic, breast, ovarian, prostate, and bladder cancers (Tables 1 and 2).

Gender-stratified analysis showed Vietnamese females had lower mortality from total cancers, esophageal cancer, breast cancer, ovarian cancer, and multiple myeloma but no significant difference in cervical and uterine cancers compared to Asian American females. Vietnamese males had higher mortality from total cancers, esophageal, stomach, laryngeal, non-Hodgkin lymphoma, and unspecified or other cancers but lower mortality from prostate cancer compared to Asian American males (Tables 1 and 2).

Age-stratified analysis revealed higher mortality among Vietnamese for total cancers and tracheal, bronchial, and lung cancers (ages 35–74), liver cancer (ages 35–84), and colorectal, stomach, pancreatic, cervical, and uterine cancers (ages 45–54), colorectal cancer (ages 45–74), among others, compared to the aggregated Asian American group. Conversely, Vietnamese had lower mortality from breast cancer (above age 55), pancreatic cancer (ages 65–74), esophageal and ovarian cancers (ages 75–84), and various cancers (above age 85) compared to aggregated Asian Americans (Tables 1 and 2).

### Other Asians

Finally, 20.37% (*n*=9,301/45,651) Asian Americans of other racial and ethnic groups died of cancer, with a mean age of death of 66.60 (SD=0.72) years, and approximately half (4,530/9,301; 48.70%) of identified as female (Table 1). Compared to the aggregated Asian American reference group, these other Asian subgroups had a higher mortality rate from pancreatic cancer but lower rates of total cancers, tracheal, bronchial, and lung cancers, liver and intrahepatic bile duct cancers, prostate cancer, bladder cancer, non-Hodgkin lymphoma, leukemia, multiple myeloma, and unspecified or other cancers (Tables 1 and 2).

Gender-stratified analysis showed other Asian females had lower mortality from esophageal cancer but no significant difference in other cancers compared to Asian American females. Other Asian males had lower mortality from prostate cancer compared to Asian American males (Tables 1 and 2). Age-stratified analysis indicated that compared to aggregated Asian Americans, other Asians were more likely to die from certain cancers at specific age intervals for tracheal, bronchial, and lung cancers, cervical and uterine cancers, and lip, oral, and pharyngeal cancers (ages 35–44); liver and intrahepatic bile duct cancers (ages 35–74), stomach and unspecified or other cancers (below age 54), total and colorectal cancers (below age 64), and bladder cancer (ages 65–74). Conversely, other Asians were less likely to die from various cancers at older ages compared to aggregated Asian Americans (Tables 1 and 2).

## Discussion

This comprehensive, national cross-sectional study examined 22 types of cancers and neoplasms among 327,311 Asian American decedents from diverse racial and ethnic backgrounds, providing critical insights for shaping future health interventions, cancer screenings, clinical care delivery, and public health efforts.

Our findings reveal consistent patterns in cancer disparities, emphasizing the need for targeted interventions tailored to high-risk populations. Notably, we noted a higher proportional mortality for most cancer types among Chinese individuals relative to aggregated Asian Americans. In contrast, Japanese individuals consistently exhibit lower proportional mortality rates (except for esophageal, pancreatic, and bladder cancers), as do "other Asian" subgroups (except for pancreatic cancer). Further, our study demonstrates that Chinese, Korean, and Vietnamese individuals face a higher likelihood of mortality from total cancers compared to aggregated Asian Americans, while Asian Indians, Japanese, and other Asians are less likely to experience such outcomes. Additionally, our investigation also found elevated proportions of stomach, colorectal, and liver, as well as intrahepatic bile duct cancer deaths among Koreans, while lower proportions were noted among Asian Indians, Filipinos, and Japanese, compared to aggregated Asian Americans.

Future epidemiological studies should aim to further characterize the complex interplay of biopsychosocial interactions and cultural factors that influence these relationships. For instance, a prior meta-analysis identified a positive association between higher levels of Kimchi consumption and risk of stomach cancer [22], which is consistent with our findings, wherein Koreans exhibited the highest proportional mortality from stomach cancers. Therefore, further emphasis should be placed on enhancing cancer screening initiatives within communities densely populated by US Korean individuals, along with the optimized allocation of resources, including primary care providers, oncologists, and educational outreach programs, to effectively address the specific healthcare needs of these communities [23, 24]. Also aligning with our study’s findings, a previous observational study showed higher rates of liver cancer deaths among Vietnamese populations, potentially attributable to the higher prevalence of hepatitis B virus (HBV) among foreign-born individuals from Vietnam [25]. Vietnam is recognized as one of the highest-risk regions globally for HBV, a risk factor for common liver cancers like hepatocellular carcinoma (HCC) [25]. As less than 50% of at-risk Asian Americans are aware of their HBV status, this further highlights the importance and urgency for greater prevention and surveillance efforts within Vietnamese and other communities that may be affected by cancers endemic to their country-of-origin. [13]

Additionally, our gender-stratified analyses reveal notable disparities in breast, cervical, and uterine deaths, with Filipina women exhibiting higher proportions when compared to the aggregated statistics for Asian American women; in contrast, Korean and Vietnamese women demonstrated lower proportions. Notably, a prior study identified a higher incidence of breast cancer among Filipina American women as compared to other Asian American groups [26]. This discrepancy was hypothesized to be attributed to specific risk factors, with cumulative menstrual and body size factors playing crucial roles for Filipina women in LA County. In contrast, Chinese women showed menstrual months alone as significant risk factors, while Japanese women identified only body size factors as contributing to their risk [26]. This highlights the crucial need for culturally-tailored approaches in health education, cancer prevention, and screening initiatives tailored specifically to diverse Asian American subgroups.

Moreover, prior research has consistently indicated that, despite the rising incidence of breast and cervical cancers among Asian American women, the uptake of screening services such as mammography and Pap smears remains notably lower compared to non-Hispanic White women [27, 28]. Further, the rates of breast and cervical screenings exhibit considerable heterogeneity across Asian American subpopulations, driven by socioeconomic factors such as disparities in access to health insurance, poverty levels, and consistent access to primary care providers [27, 28]. These variations extend to differences in the epidemiology of breast and cervical cancer subtypes, influencing prognoses and treatment approaches [29].

For example, the heightened prevalence of HER2/neu-positive breast cancers among Filipina women in contrast to other Asian American subpopulations may contribute to an increased risk of breast cancer-related morbidity and mortality [29, 30]. This aligns with the findings of our study, indicating that Filipina women demonstrated the highest proportional mortality from breast cancer.

Within the broader perspective, our study reinforces the urgent call for enhanced racial and ethnic data disaggregation among Asian American subpopulations. Our findings highlight significant cancer disparities, challenging the oversimplified notion of a generally lower relative risk of cancer among "Asian Americans" compared to non-Hispanic White populations when conducting aggregated data analyses [29, 31]. Collecting and reporting such nuanced data is vital in addressing health disparities, facilitating targeted interventions, and promoting health equity. For instance, in the clinical setting, clinicians may choose to provide patients with more autonomy when collecting information on racial and ethnic identities, in addition to data administrators and hospital leaders ensuring digital infrastructure and Electronic Medical Record (EMR) systems are optimized to accommodate and accurately capture the diversity of racial and ethnic identities [32]. These measures to improve the granularity of racial and ethnic data are imperative to reduce misclassification, counteract bias and discrimination, and inform tailored clinical and public health interventions for Asian American patients [29].

Further, within the domains of public health and community health, there needs to be an increasing demand for federal, state, and institutional agencies to enhance disease surveillance and demographic coding within databases to improve the precision of racial and ethnic data. For instance, the recent mandate by organizations such as the Centers for Medicare & Medicaid Services (CMS) to include more comprehensive sets of disaggregated racial and ethnic categories within Medicare Advantage and Medicare prescription drug (Part D) plans, starting January 2023, is a positive step forward [11]. Nevertheless, most federal agencies still adhere to the minimum reporting standards set by the OMB, for which the classifications are limited to “Asian” and “Native Hawaiian or Other Pacific Islander”, presenting a major impediment to understanding racial and ethnic disparities in cancer mortality and other causes of death among Asian American populations [11]. Some federal datasets with disaggregated racial and ethnic data on Asian American subgroups have newly imposed restrictions on public access [33]. These outdated data collection practices threatens to perpetuate the obscuration of critical health disparities, if left unrectified.

Researchers and community-based organizations can also play vital roles by collecting data and designing surveys with more precise disaggregated racial and ethnic categories, in addition to supporting disaggregated data analysis practices that accurately represent historically excluded populations and advance health disparities research [29]. Community-based participatory research and collaborations with local organizations may also prove essential to tailor data collection methods to local Asian American demographics and cultural contexts, guiding policymakers and healthcare providers toward effective interventions for risk prevention (e.g., reducing tobacco usage), improving cancer screening, and other equitable health outcomes [34–36].

For instance, a previous qualitative study conducted among Filipino men in Hawaii identified barriers to prostate cancer screening, including a lack of awareness and knowledge, negative beliefs and fears, as well as a tendency to seeking healthcare only when symptoms became apparent—similar to trends observed in other ethnic and racial minority groups [36]. However, the Filipino population in Hawaii presented distinct challenges specific to prostate cancer screening. Notably, male heads of households diagnosed with prostate cancer were perceived as burdens to their families, which may have contributed to the prevalent inclination to postpone recommended healthcare and cancer screenings [36]. Therein lies a clinical imperative to implement targeted education and culturally-sensitive training for Filipino healthcare providers and community health leaders with regard to their roles in promoting prostate cancer screening. [26]

Finally, in light of significant social determinants of health—including health literacy challenges, language barriers, and limited access to health insurance prevalent among Asian Americans, particularly Asian immigrants to the US—there is a need to enhance current data visualization and reporting strategies to advancing health equity [37]. While overall limited English proficiency is estimated at around 35% among Asian Americans, there is considerable heterogeneity among subgroups, with the highest prevalence among Vietnamese (53%), Chinese (46%), and Korean (45%) subgroups [28]. Collaborative efforts with community-based organizations become pivotal in optimizing cancer screening initiatives informed by disaggregated data analyses. This approach ensures not only the effective knowledge translation but may also foster equitable access to cancer screening and care, aiming to reduce cancer morbidity and mortality among at-risk Asian American subpopulations.

### Limitations

Several limitations warrant consideration in interpreting our study findings. Firstly, our cross-sectional design restricts our ability to establish causal relationships between observed disparities and factors contributing to deaths due to cancers and neoplasms within Asian American subpopulations. Moreover, we analyzed deaths from these cancers as an underlying cause of death. While this approach may yield more precise estimates of deaths directly linked to various cancer types, it may come at the expense of potentially underreporting cancer-related deaths in this sample and a higher risk of misclassification. More broadly, our reliance on death certificate data may be subject to miscoding of the causes of death, racial misclassification, and excludes other racial and ethnic Asian American subpopulations not listed on these death certificates. For instance, our reliance on the CDC WONDER database limited our ability to analyze critical health disparities among specific Asian ethnic groups, such as Thai, Cambodian, Hmong, Laotian, and others. Many of these groups had smaller sample sizes and were thus categorized under 'other Asians' in the CDC WONDER database. Further, our study findings should only be generalized to Asian individuals living in the US. Secondly, given the objective of this study to broadly overview and identify disparities in cancer mortality, we did not link CDC WONDER data with other social and demographic factors such as migrant status, socioeconomic status, access to health services, and more. Future research could explore additional clinical factors such as stage at diagnosis, specific comorbidities, medications and therapies received, health insurance status, and vaccinations (e.g., Hepatitis B vaccination, given the relatively high liver cancer mortality in many Asian American subgroups). Further, due to substantial amounts of data suppression in the CDC WONDER database with respect to disaggregated NHPI data, we omitted these subgroups from our analyses. Future studies using other cancer databases should aim to conduct a comprehensive evaluation of mortality trends among NHPI subgroups. Future studies evaluating cancer mortality disparities among disaggregated NHPI subgroups may consider other databases including the California Health Interview Survey (CHIS); Surveillance, Epidemiology, and End Results (SEER), National Cancer Database, and others. Additionally, future studies should incorporate qualitative and individual-level data to provide a deeper understanding of individuals’ lived experiences and lifestyle or environmental factors contributing to these disparities, thereby enhancing the overall interpretative framework of cancer-related mortality among Asian American and NHPI populations.

## Conclusion

In summary, our study highlights the critical importance of implementing targeted cancer prevention, treatment, and surveillance initiatives tailored to specific Asian American subgroups, given the nuanced patterns of cancer mortality observed. The amalgamation of racial and ethnic data masks significant variations in cancer mortality risk within diverse AANHPI populations. Our findings underscore the urgent need for improving disaggregated racial and ethnic data collection and reporting, challenging prevailing misconceptions associated with the "model minority myth", and laying the foundation for targeted social and health interventions that can effectively address disparities in cancer mortality among AANHPI populations.

## Supplementary Information

Below is the link to the electronic supplementary material.

Supplementary file1 (DOCX 32 KB)

Supplementary file2 (DOCX 33 KB)

Supplementary file3 (DOCX 43 KB)

## Data Availability

All data for this study are publicly available at https://wonder.cdc.gov/Deaths-by-Underlying-Cause.html.
