# Supplementary material for: Disparities in Cancer Mortality among Disaggregated Asian American Subpopulations, 2018–2021
Source: J Racial Ethn Health Disparities. 2024 Jun 25;12(4):2473–95. doi: 10.1007/s40615-024-02067-0 (PMC12241243; doi:10.1007/s40615-024-02067-0)
Supplement: Supplementary file 2 — Supplementary file2 (DOCX 33 KB) [file 40615_2024_2067_MOESM2_ESM.docx]

**Table S2.** Native Hawaiian and Pacific Islander digestive and respiratory cancer deaths, 2018-2021

| **Cause of death** | **Aggregate NHPI^1^** | **Hawaiian** | **Guamanian** | **Samoan** | **Other Pacific Islander** |
| --- | --- | --- | --- | --- | --- |
| Total decedents | 17,845  (100.00) | 3,940  (100.00) | 2,205  (100.00) | 4,187  (100.00) | 7,513  (100.00) |
| **Total cancers** | | | | | |
| **Overall (N, %)** | 3284 (18.40) | 811 (20.58) | 368 (16.69) | 749 (17.89) | 1356 (18.05) |
| **Mean age of death (years, SD)** | 64.43 (0.74) | — | — | — | — |
| **Sex** |  |  |  |  |  |
| Female (N, %) | 1722 (9.65) | 421 (10.69) | 175 (7.94) | 419 (10.01) | 707 (9.41) |
| Male (N, %) | 1562 (8.75) | 390 (9.90) | 193 (8.75) | 330 (7.88) | 649 (8.64) |
| Suppressed or sex not stated (N, %) | 0 (0.00) | 0 (0.00) | 0 (0.00) | 0 (0.00) | 0 (0.00) |
| **Age** |  |  |  |  |  |
| <35 years (N, %) | 108 (0.61) | — | — | 14 (0.33) | 59 (0.79) |
| 35-44 years (N, %) | 222 (1.24) | 20 (0.51) | 20 (0.91) | 60 (1.43) | 122 (1.62) |
| 45-54 years (N, %) | 438 (2.45) | 60 (1.52) | 50 (2.27) | 116 (2.77) | 212 (2.82) |
| 55-64 years (N, %) | 806 (4.52) | 183 (4.64) | 97 (4.40) | 184 (4.39) | 342 (4.55) |
| 65-74 years (N, %) | 949 (5.32) | 261 (6.62) | 103 (4.67) | 227 (5.42) | 358 (4.77) |
| 75-84 years (N, %) | 535 (3.00) | 185 (4.70) | 60 (2.72) | 110 (2.63) | 180 (2.40) |
| ≥85 years (N, %) | 216 (1.21) | 89 (2.26) | 30 (1.36) | 28 (0.67) | 69 (0.92) |
| Suppressed or age not stated (N, %) | 10 (0.06) | 13 (0.33) | 8 (0.36) | 10 (0.24) | 14 (0.19) |
| **Lip, oral, and pharyngeal cancers** | | | | | |
| **Overall (N, %)** | 79 (0.44) | 17 (0.43) | — | 10 (0.24) | 45 (0.60) |
| **Mean age of death (years, SD)** | — | — | — | — | — |
| **Sex** |  |  |  |  |  |
| Female (N, %) | 24 (0.13) | — | — | — | 12 (0.16) |
| Male (N, %) | 55 (0.31) | 11 (0.28) | — | — | 33 (0.44) |
| Suppressed or sex not stated (N, %) | 0 (0.00) | 6 (0.15) | 0 (0.00) | 10 (0.24) | 0 (0.00) |
| **Age** |  |  |  |  |  |
| <35 years (N, %) | — | — | — | — | — |
| 35-44 years (N, %) | — | — | — | — | — |
| 45-54 years (N, %) | 16 (0.09) | — | — | — | 10 (0.13) |
| 55-64 years (N, %) | 21 (0.12) | — | — | — | 10 (0.13) |
| 65-74 years (N, %) | 19 (0.11) | — | — | — | 11 (0.15) |
| 75-84 years (N, %) | — | — | — | — | — |
| ≥85 years (N, %) | — | — | — | — | — |
| Suppressed or sex not stated (N, %) | 23 (0.13) | 17 (0.43) | 0 (0.00) | 10 (0.24) | 14 (0.19) |
| **Esophageal cancers** | | | | | |
| **Overall (N, %)** | 48 (0.27) | 21 (0.53) | — | — | 14 (0.19) |
| **Mean age of death (years, SD)** | — | — | — | — | — |
| **Sex** |  |  |  |  |  |
| Female (N, %) | 12 (0.07) | — | — | — | — |
| Male (N, %) | 36 (0.20) | 16 (0.41) | — | — | — |
| Suppressed or sex not stated (N, %) | 0 (0.00) | 5 (0.13) | 0 (0.00) | 0 (0.00) | 14 (0.19) |
| **Age** |  |  |  |  |  |
| <35 years (N, %) | — | — | — | — | — |
| 35-44 years (N, %) | — | — | — | — | — |
| 45-54 years (N, %) | — | — | — | — | — |
| 55-64 years (N, %) | 14 (0.08) | — | — | — | — |
| 65-74 years (N, %) | 20 (0.11) | — | — | — | — |
| 75-84 years (N, %) | — | — | — | — | — |
| ≥85 years (N, %) | — | — | — | — | — |
| Suppressed or age not stated (N, %) | 14 (0.08) | 21 (0.53) | 0 (0.00) | 0 (0.00) | 14 (0.19) |
| **Stomach cancers** | | | | | |
| **Overall (N, %)** | 138 (0.77) | 16 (0.41) | 14 (0.63) | 49 (1.17) | 59 (0.79) |
| **Mean age of death (years, SD)** | — | — | — | — | — |
| **Sex** |  |  |  |  |  |
| Female (N, %) | 54 (0.30) | — | — | 21 (0.50) | 23 (0.31) |
| Male (N, %) | 84 (0.47) | 13 (0.33) | — | 28 (0.67) | 36 (0.48) |
| Suppressed or sex not stated (N, %) | 0 (0.00) | 3 (0.08) | 14 (0.63) | 0 (0.00) | 0 (0.00) |
| **Age** |  |  |  |  |  |
| <35 years (N, %) | — | — | — | — | — |
| 35-44 years (N, %) | 14 (0.08) | — | — | — | 10 (0.13) |
| 45-54 years (N, %) | 23 (0.13) | — | — | — | 12 (0.16) |
| 55-64 years (N, %) | 32 (0.18) | — | — | 14 (0.33) | 13 (0.17) |
| 65-74 years (N, %) | 32 (0.18) | — | — | 14 (0.33) | 12 (0.16) |
| 75-84 years (N, %) | 24 (0.13) | — | — | 10 (0.24) | — |
| ≥85 years (N, %) | — | — | — | — | — |
| Suppressed or age not stated (N, %) | 13 (0.07) | 16 (0.41) | 14 (0.63) | 11 (0.26) | 12 (0.16) |
| **Colon, rectal, and anal cancers** | | | | | |
| **Overall (N, %)** | 274 (1.54) | 73 (1.85) | 34 (1.54) | 60 (1.43) | 107 (1.42) |
| **Mean age of death (years, SD)** | 58.54 (3.02) | — | — | — | — |
| **Sex** |  |  |  |  |  |
| Female (N, %) | 124 (0.69) | 32 (0.81) | 15 (0.68) | 25 (0.60) | 52 (0.69) |
| Male (N, %) | 150 (0.84) | 41 (1.04) | 19 (0.86) | 35 (0.84) | 55 (0.73) |
| Suppressed or sex not stated (N, %) | 0 (0.00) | 0 (0.00) | 0 (0.00) | 0 (0.00) | 0 (0.00) |
| **Age** |  |  |  |  |  |
| <35 years (N, %) | 12 (0.07) | — | — | — | — |
| 35-44 years (N, %) | 29 (0.16) | — | — | 11 (0.26) | — |
| 45-54 years (N, %) | 57 (0.32) | 14 (0.36) | — | 14 (0.33) | 25 (0.33) |
| 55-64 years (N, %) | 76 (0.43) | 22 (0.56) | 10 (0.45) | 13 (0.31) | 31 (0.41) |
| 65-74 years (N, %) | 53 (0.30) | 17 (0.43) | — | 10 (0.24) | 19 (0.25) |
| 75-84 years (N, %) | 27 (0.15) | — | — | — | 10 (0.13) |
| ≥85 years (N, %) | 18 (0.10) | — | — | — | — |
| Suppressed or age not stated (N, %) | 2 (0.01) | 20 (0.51) | 24 (1.09) | 12 (0.29) | 22 (0.29) |
| **Liver and intrahepatic bile duct cancers** | | | | | |
| **Overall (N, %)** | 251 (1.41) | 48 (1.22) | 30 (1.36) | 36 (0.86) | 137 (1.82) |
| **Mean age of death (years, SD)** | 64.76 (2.87) | — | — | — | — |
| **Sex** |  |  |  |  |  |
| Female (N, %) | 76 (0.43) | 15 (0.38) | 13 (0.59) | 13 (0.31) | 35 (0.47) |
| Male (N, %) | 175 (0.98) | 33 (0.84) | 17 (0.77) | 23 (0.55) | 102 (1.36) |
| Suppressed or sex not stated (N, %) | 0 (0.00) | 0 (0.00) | 0 (0.00) | 0 (0.00) | 0 (0.00) |
| **Age** |  |  |  |  |  |
| <35 years (N, %) | — | — | — | — | — |
| 35-44 years (N, %) | 27 (0.15) | — | — | — | 23 (0.31) |
| 45-54 years (N, %) | 38 (0.21) | — | — | — | 20 (0.27) |
| 55-64 years (N, %) | 74 (0.41) | 11 (0.28) | 10 (0.45) | 15 (0.36) | 38 (0.51) |
| 65-74 years (N, %) | 66 (0.37) | 11 (0.28) | — | 12 (0.29) | 37 (0.49) |
| 75-84 years (N, %) | 32 (0.18) | 14 (0.36) | — | — | 14 (0.19) |
| ≥85 years (N, %) | 12 (0.07) | — | — | — | — |
| Suppressed or age not stated (N, %) | 2 (0.01) | 12 (0.30) | 20 (0.91) | 9 (0.21) | 5 (0.07) |
| **Pancreatic cancers** | | | | | |
| **Overall (N, %)** | 217 (1.22) | 68 (1.73) | 20 (0.91) | 46 (1.10) | 83 (1.10) |
| **Mean age of death (years, SD)** | 66.25 (3.25) | — | — | — | — |
| **Sex** |  |  |  |  |  |
| Female (N, %) | 120 (0.67) | 36 (0.91) | — | 28 (0.67) | 48 (0.64) |
| Male (N, %) | 97 (0.54) | 32 (0.81) | 12 (0.54) | 18 (0.43) | 35 (0.47) |
| Suppressed or sex not stated (N, %) | 0 (0.00) | 0 (0.00) | 8 (0.36) | 0 (0.00) | 0 (0.00) |
| **Age** |  |  |  |  |  |
| <35 years (N, %) | — | — | — | — | — |
| 35-44 years (N, %) | — | — | — | — | — |
| 45-54 years (N, %) | 30 (0.17) | — | — | — | 13 (0.17) |
| 55-64 years (N, %) | 51 (0.29) | 17 (0.43) | — | 11 (0.26) | 19 (0.25) |
| 65-74 years (N, %) | 68 (0.38) | 16 (0.41) | — | 16 (0.38) | 28 (0.37) |
| 75-84 years (N, %) | 48 (0.27) | 23 (0.58) | — | — | 16 (0.21) |
| ≥85 years (N, %) | 13 (0.07) | — | — | — | — |
| Suppressed or age not stated (N, %) | 7 (0.04) | 12 (0.30) | 20 (0.91) | 19 (0.45) | 7 (0.09) |
| **Laryngeal cancers** | | | | | |
| **Overall (N, %)** | — | — | — | — | — |
| **Mean age of death (years, SD)** | — | — | — | — | — |
| **Sex** |  |  |  |  |  |
| Female (N, %) | — | — | — | — | — |
| Male (N, %) | — | — | — | — | — |
| Suppressed or sex not stated (N, %) | — | — | — | — | — |
| **Age** |  |  |  |  |  |
| <35 years (N, %) | — | — | — | — | — |
| 35-44 years (N, %) | — | — | — | — | — |
| 45-54 years (N, %) | — | — | — | — | — |
| 55-64 years (N, %) | — | — | — | — | — |
| 65-74 years (N, %) | — | — | — | — | — |
| 75-84 years (N, %) | — | — | — | — | — |
| ≥85 years (N, %) | — | — | — | — | — |
| Suppressed or age not stated (N, %) | — | — | — | — | — |
| **Tracheal, bronchial, and lung cancers** | | | | | |
| **Overall (N, %)** | 588 (3.30) | 177 (4.49) | 79 (3.58) | 131 (3.13) | 201 (2.68) |
| **Mean age of death (years, SD)** | 69.76 (1.19) | — | — | — | — |
| **Sex** |  |  |  |  |  |
| Female (N, %) | 261 (1.46) | 95 (2.41) | 31 (1.41) | 47 (1.12) | 88 (1.17) |
| Male (N, %) | 327 (1.83) | 82 (2.08) | 48 (2.18) | 84 (2.01) | 113 (1.50) |
| Suppressed or sex not stated (N, %) | 0 (0.00) | 0 (0.00) | 0 (0.00) | 0 (0.00) | 0 (0.00) |
| **Age** |  |  |  |  |  |
| <35 years (N, %) | — | — | — | — | — |
| 35-44 years (N, %) | 12 (0.07) | — | — | — | — |
| 45-54 years (N, %) | 40 (0.22) | — | — | 15 (0.36) | 18 (0.24) |
| 55-64 years (N, %) | 147 (0.82) | 35 (0.89) | 23 (1.04) | 32 (0.76) | 57 (0.76) |
| 65-74 years (N, %) | 212 (1.19) | 71 (1.80) | 29 (1.32) | 51 (1.22) | 61 (0.81) |
| 75-84 years (N, %) | 127 (0.71) | 49 (1.24) | 17 (0.77) | 24 (0.57) | 37 (0.49) |
| ≥85 years (N, %) | 46 (0.26) | 19 (0.48) | — | — | 15 (0.20) |
| Suppressed or age not stated (N, %) | 4 (0.02) | 3 (0.08) | 10 (0.45) | 9 (0.21) | 13 (0.17) |

**Note:** All percentages are calculated using total decedents in each racial/ethnic subgroup as the denominator

**^1^**NHPI, Native Hawaiian and Pacific Islander

— indicates suppressed data values
