# Supplementary material for: Disparities in Cancer Mortality among Disaggregated Asian American Subpopulations, 2018–2021
Source: J Racial Ethn Health Disparities. 2024 Jun 25;12(4):2473–95. doi: 10.1007/s40615-024-02067-0 (PMC12241243; doi:10.1007/s40615-024-02067-0)
Supplement: Supplementary file 3 — Supplementary file3 (DOCX 43 KB) [file 40615_2024_2067_MOESM3_ESM.docx]

**Table S3.** Native Hawaiian and Pacific Islander reproductive, urinary, and hematopoietic, lymphatic, and other cancer deaths, 2018-2021

| **Cause of death** | **Aggregate NHPI^1^** | **Hawaiian** | **Guamanian** | **Samoan** | **Other Pacific Islander** |
| --- | --- | --- | --- | --- | --- |
| **Total decedents** | 17,845  (100.00) | 3,940  (100.00) | 2,205  (100.00) | 4,187  (100.00) | 7,513  (100.00) |
| **Skin cancers** | | | | | |
| **Overall (N, %)** | 12 (0.07) | — | — | — | — |
| **Mean age of death (years, SD)** | — | — | — | — | — |
| **Sex** |  |  |  |  |  |
| Female (N, %) | — | — | — | — | — |
| Male (N, %) | — | — | — | — | — |
| Suppressed or sex not stated (N, %) | 12 (0.07) | — | — | — | — |
| **Age** |  |  |  |  |  |
| <35 years (N, %) | — | — | — | — | — |
| 35-44 years (N, %) | — | — | — | — | — |
| 45-54 years (N, %) | — | — | — | — | — |
| 55-64 years (N, %) | — | — | — | — | — |
| 65-74 years (N, %) | — | — | — | — | — |
| 75-84 years (N, %) | — | — | — | — | — |
| ≥85 years (N, %) | — | — | — | — | — |
| Suppressed or age not stated (N, %) | 12 (0.07) | — | — | — | — |
| **Breast cancers** | | | | | |
| **Overall (N, %)** | 320 (1.79) | 65 (1.65) | 44 (2.00) | 89 (2.13) | 122 (1.62) |
| **Mean age of death (years, SD)** | 62.26 (1.90) | — | — | — | — |
| **Sex** |  |  |  |  |  |
| Female (N, %) | 318 (1.78) | 64 (1.62) | 44 (2.00) | 88 (2.10) | 122 (1.62) |
| Male (N, %) | — | — | — | — | — |
| Suppressed or sex not stated (N, %) | 2 (0.01) | 1 (0.03) | — | 1 (0.02) | — |
| **Age** |  |  |  |  |  |
| <35 years (N, %) | — | — | — | — | — |
| 35-44 years (N, %) | 34 (0.19) | — | — | 10 (0.24) | 15 (0.20) |
| 45-54 years (N, %) | 54 (0.30) | — | — | 14 (0.33) | 25 (0.33) |
| 55-64 years (N, %) | 92 (0.52) | 16 (0.41) | 11 (0.50) | 27 (0.64) | 38 (0.51) |
| 65-74 years (N, %) | 83 (0.47) | 23 (0.58) | 11 (0.50) | 24 (0.57) | 25 (0.33) |
| 75-84 years (N, %) | 39 (0.22) | 10 (0.25) | — | 10 (0.24) | 12 (0.16) |
| ≥85 years (N, %) | 16 (0.09) | — | — | — | — |
| Suppressed or age not stated (N, %) | 2 (0.01) | 16 (0.41) | 22 (1.00) | 4 (0.10) | 7 (0.09) |
| **Cervical and uterine cancers** | | | | | |
| **Overall (N, %)** | 253 (1.42) | 54 (1.37) | — | 82 (1.96) | 114 (1.52) |
| **Mean age of death (years, SD)** | — | — | — | — | — |
| **Sex** |  |  |  |  |  |
| Female (N, %) | 253 (1.42) | 35 (0.89) | — | 82 (1.96) | 114 (1.52) |
| Male (N, %) | 0 (0.00) | 0 (0.00) | — | 0 (0.00) | 0 (0.00) |
| Suppressed or sex not stated (N, %) | 0 (0.00) | 19 (0.48) | — | 0 (0.00) | 0 (0.00) |
| **Age** |  |  |  |  |  |
| <35 years (N, %) | — | — | — | — | — |
| 35-44 years (N, %) | 31 (0.17) | — | — | 10 (0.24) | 10 (0.13) |
| 45-54 years (N, %) | 60 (0.34) | — | — | 20 (0.48) | 16 (0.21) |
| 55-64 years (N, %) | 60 (0.34) | 10 (0.25) | — | 13 (0.31) | 21 (0.28) |
| 65-74 years (N, %) | 68 (0.38) | 13 (0.33) | — | 18 (0.43) | 21 (0.28) |
| 75-84 years (N, %) | 16 (0.09) | — | — | — | — |
| ≥85 years (N, %) | — | — | — | — | — |
| Suppressed or age not stated (N, %) | 18 (0.10) | 31 (0.79) | — | 21 (0.50) | 46 (0.61) |
| **Ovarian cancers** | | | | | |
| **Overall (N, %)** | 65 (0.36) | 19 (0.48) | 0 (0.00) | 18 (0.43) | 22 (0.29) |
| **Mean age of death (years, SD)** | — | — | — | — | — |
| **Sex** |  |  |  |  |  |
| Female (N, %) | 65 (0.36) | 19 (0.48) | — | 18 (0.43) | 22 (0.29) |
| Male (N, %) | 0 (0.00) | 0 (0.00) | — | 0 (0.00) | 0 (0.00) |
| Suppressed or sex not stated (N, %) | 0 (0.00) | 0 (0.00) | — | 0 (0.00) | 0 (0.00) |
| **Age** |  |  |  |  |  |
| <35 years (N, %) | — | — | — | — | — |
| 35-44 years (N, %) | — | — | — | — | — |
| 45-54 years (N, %) | 15 (0.08) | — | — | — | — |
| 55-64 years (N, %) | 14 (0.08) | — | — | — | — |
| 65-74 years (N, %) | 17 (0.10) | — | — | — | — |
| 75-84 years (N, %) | — | — | — | — | — |
| ≥85 years (N, %) | — | — | — | — | — |
| Suppressed or age not stated (N, %) | 19 (0.11) | 19 (0.48) | — | 18 (0.43) | 22 (0.29) |
| **Prostate cancers** | | | | | |
| **Overall (N, %)** | 150 (0.84) | 28 (0.71) | 19 (0.86) | 43 (1.03) | 60 (0.80) |
| **Mean age of death (years, SD)** | 72.00 (7.16) | — | — | — | — |
| **Sex** |  |  |  |  |  |
| Female (N, %) | 0 (0.00) | 0 (0.00) | 0 (0.00) | 0 (0.00) | 0 (0.00) |
| Male (N, %) | 150 (0.84) | 28 (0.71) | 19 (0.86) | 43 (1.03) | 60 (0.80) |
| Suppressed or sex not stated (N, %) | 0 (0.00) | 0 (0.00) | 0 (0.00) | 0 (0.00) | 0 (0.00) |
| **Age** |  |  |  |  |  |
| <35 years (N, %) | — | — | — | — | — |
| 35-44 years (N, %) | — | — | — | — | — |
| 45-54 years (N, %) | — | — | — | — | — |
| 55-64 years (N, %) | 19 (0.11) | — | — | — | — |
| 65-74 years (N, %) | 55 (0.31) | 11 (0.28) | — | 14 (0.33) | 25 (0.33) |
| 75-84 years (N, %) | 47 (0.26) | — | — | 19 (0.45) | 16 (0.21) |
| ≥85 years (N, %) | 26 (0.15) | — | — | — | 12 (0.16) |
| Suppressed or age not stated (N, %) | 3 (0.02) | 17 (0.43) | 19 (0.86) | 10 (0.24) | 7 (0.09) |
| **Kidney cancers** | | | | | |
| **Overall (N, %)** | 54 (0.30) | 17 (0.43) | 10 (0.45) | 11 (0.26) | 21 (0.28) |
| **Mean age of death (years, SD)** | — | — | — | — | — |
| **Sex** |  |  |  |  |  |
| Female (N, %) | 19 (0.11) | — | — | — | — |
| Male (N, %) | 35 (0.20) | 12 (0.30) | — | — | 14 (0.19) |
| Suppressed or sex not stated (N, %) | — | 5 (0.13) | 10 (0.45) | 11 (0.26) | 7 (0.09) |
| **Age** |  |  |  |  |  |
| <35 years (N, %) | — | — | — | — | — |
| 35-44 years (N, %) | — | — | — | — | — |
| 45-54 years (N, %) | — | — | — | — | — |
| 55-64 years (N, %) | 13 (0.07) | — | — | — | — |
| 65-74 years (N, %) | 19 (0.11) | — | — | — | — |
| 75-84 years (N, %) | — | — | — | — | — |
| ≥85 years (N, %) | — | — | — | — | — |
| Suppressed or age not stated (N, %) | 22 (0.12) | 17 (0.43) | 10 (0.45) | 11 (0.26) | 21 (0.28) |
| **Bladder cancers** | | | | | |
| **Overall (N, %)** | 49 (0.27) | 17 (0.43) | 26 (1.18) | — | 17 (0.23) |
| **Mean age of death (years, SD)** | — | — | — | — | — |
| **Sex** |  |  |  |  |  |
| Female (N, %) | 14 (0.08) | — | — | — | — |
| Male (N, %) | 35 (0.20) | — | — | — | 13 (0.17) |
| Suppressed or sex not stated (N, %) | 0 (0.00) | 17 (0.43) | 26 (1.18) | 0 (0.00) | 4 (0.05) |
| **Age** |  |  |  |  |  |
| <35 years (N, %) | — | — | — | — | — |
| 35-44 years (N, %) | — | — | — | — | — |
| 45-54 years (N, %) | — | — | — | — | — |
| 55-64 years (N, %) | — | — | — | — | — |
| 65-74 years (N, %) | 12 (0.07) | — | — | — | — |
| 75-84 years (N, %) | 14 (0.08) | — | — | — | — |
| ≥85 years (N, %) | 13 (0.07) | — | — | — | — |
| Suppressed or age not stated (N, %) | 10 (0.06) | 17 (0.43) | 26 (1.18) | 0 (0.00) | 17 (0.23) |
| **Brain, meningeal, or other central nervous system cancers** | | | | | |
| **Overall (N, %)** | 73 (0.41) | 11 (0.28) | 10 (0.45) | 11 (0.26) | 41 (0.55) |
| **Mean age of death (years, SD)** | — | — | — | — | — |
| **Sex** |  |  |  |  |  |
| Female (N, %) | 39 (0.22) | — | — | — | 24 (0.32) |
| Male (N, %) | 34 (0.19) | — | — | — | 17 (0.23) |
| Suppressed or sex not stated (N, %) | 0 (0.00) | 11 (0.28) | 10 (0.45) | 11 (0.26) | 0 (0.00) |
| **Age** |  |  |  |  |  |
| <35 years (N, %) | — | — | — | — | — |
| 35-44 years (N, %) | — | — | — | — | — |
| 45-54 years (N, %) | 10 (0.06) | — | — | — | — |
| 55-64 years (N, %) | 16 (0.09) | — | — | — | — |
| 65-74 years (N, %) | 17 (0.10) | — | — | — | — |
| 75-84 years (N, %) | — | — | — | — | — |
| ≥85 years (N, %) | — | — | — | — | — |
| Suppressed or age not stated (N, %) | 30 (0.17) | 11 (0.28) | 10 (0.45) | 11 (0.26) | 41 (0.55) |
| **Hodgkin disease** | | | | | |
| **Overall (N, %)** | — | — | — | — | — |
| **Mean age of death (years, SD)** | — | — | — | — | — |
| **Sex** |  |  |  |  |  |
| Female (N, %) | — | — | — | — | — |
| Male (N, %) | — | — | — | — | — |
| Suppressed or sex not stated (N, %) | — | — | — | — | — |
| **Age** |  |  |  |  |  |
| <35 years (N, %) | — | — | — | — | — |
| 35-44 years (N, %) | — | — | — | — | — |
| 45-54 years (N, %) | — | — | — | — | — |
| 55-64 years (N, %) | — | — | — | — | — |
| 65-74 years (N, %) | — | — | — | — | — |
| 75-84 years (N, %) | — | — | — | — | — |
| ≥85 years (N, %) | — | — | — | — | — |
| Suppressed or age not stated (N, %) | — | — | — | — | — |
| **Non-Hodgkin lymphoma** | | | | | |
| **Overall (N, %)** | 100 (0.56) | 23 (0.58) | 16 (0.73) | 18 (0.43) | 43 (0.57) |
| **Mean age of death (years, SD)** | — | — | — | — | — |
| **Sex** |  |  |  |  |  |
| Female (N, %) | 48 (0.27) | 14 (0.36) | — | — | 23 (0.31) |
| Male (N, %) | 52 (0.29) | — | 11 (0.50) | 12 (0.29) | 20 (0.27) |
| Suppressed or sex not stated (N, %) | — | 9 (0.23) | 5 (0.23) | 6 (0.14) | — |
| **Age** |  |  |  |  |  |
| <35 years (N, %) | — | — | — | — | — |
| 35-44 years (N, %) | — | — | — | — | — |
| 45-54 years (N, %) | 11 (0.06) | — | — | — | — |
| 55-64 years (N, %) | 25 (0.14) | — | — | — | 10 (0.13) |
| 65-74 years (N, %) | 27 (0.15) | — | — | — | 12 (0.16) |
| 75-84 years (N, %) | 19 (0.11) | — | — | — | — |
| ≥85 years (N, %) | — | — | — | — | — |
| Suppressed or age not stated (N, %) | 18 (0.10) | 23 (0.58) | 16 (0.73) | 18 (0.43) | 21 (0.28) |
| **Leukemia** | | | | | |
| **Overall (N, %)** | 129 (0.72) | 39 (0.99) | — | 24 (0.57) | 59 (0.79) |
| **Mean age of death (years, SD)** | — | — | — | — | — |
| **Sex** |  |  |  |  |  |
| Female (N, %) | 55 (0.31) | 14 (0.36) | — | 12 (0.29) | 26 (0.35) |
| Male (N, %) | 74 (0.41) | 25 (0.63) | — | 12 (0.29) | 33 (0.44) |
| Suppressed or sex not stated (N, %) | 0 (0.00) | 0 (0.00) | — | 0 (0.00) | 0 (0.00) |
| **Age** |  |  |  |  |  |
| <35 years (N, %) | 11 (0.06) | — | — | — | — |
| 35-44 years (N, %) | — | — | — | — | — |
| 45-54 years (N, %) | 13 (0.07) | — | — | — | — |
| 55-64 years (N, %) | 19 (0.11) | — | — | — | — |
| 65-74 years (N, %) | 34 (0.19) | 10 (0.25) | — | — | 16 (0.21) |
| 75-84 years (N, %) | 22 (0.12) | 10 (0.25) | — | — | — |
| ≥85 years (N, %) | 13 (0.07) | — | — | — | — |
| Suppressed or age not stated (N, %) | 17 (0.10) | 19 (0.48) | — | 24 (0.57) | 43 (0.57) |
| **Multiple myeloma and immunoproliferative neoplasms** | | | | | |
| **Overall (N, %)** | 56 (0.31) | 16 (0.41) | — | — | 28 (0.37) |
| **Mean age of death (years, SD)** | — | — | — | — | — |
| **Sex** |  |  |  |  |  |
| Female (N, %) | 26 (0.15) | — | — | — | 15 (0.20) |
| Male (N, %) | 30 (0.17) | — | — | — | 13 (0.17) |
| Suppressed or sex not stated (N, %) | 0 (0.00) | 16 (0.41) | — | — | 0 (0.00) |
| **Age** |  |  |  |  |  |
| <35 years (N, %) | — | — | — | — | — |
| 35-44 years (N, %) | — | — | — | — | — |
| 45-54 years (N, %) | — | — | — | — | — |
| 55-64 years (N, %) | 17 (0.10) | — | — | — | — |
| 65-74 years (N, %) | 18 (0.10) | — | — | — | — |
| 75-84 years (N, %) | 15 (0.08) | — | — | — | — |
| ≥85 years (N, %) | — | — | — | — | — |
| Suppressed or age not stated (N, %) | 6 (0.03) | 16 (0.41) | — | — | 28 (0.37) |
| **Other and unspecified cancers** | | | | | |
| **Overall (N, %)** | 418 (2.34) | 105 (2.66) | 40 (1.81) | 97 (2.32) | 176 (2.34) |
| **Mean age of death (years, SD)** | 65.33 (1.49) | — | — | — | — |
| **Sex** |  |  |  |  |  |
| Female (N, %) | 208 (1.17) | 47 (1.19) | 17 (0.77) | 59 (1.41) | 85 (1.13) |
| Male (N, %) | 210 (1.18) | 58 (1.47) | 23 (1.04) | 38 (0.91) | 91 (1.21) |
| Suppressed or sex not stated (N, %) | 0 (0.00) | 0 (0.00) | 0 (0.00) | 0 (0.00) | 0 (0.00) |
| **Age** |  |  |  |  |  |
| <35 years (N, %) | 14 (0.08) | — | — | — | — |
| 35-44 years (N, %) | 28 (0.16) | — | — | — | 12 (0.16) |
| 45-54 years (N, %) | 50 (0.28) | — | — | 10 (0.24) | 31 (0.41) |
| 55-64 years (N, %) | 103 (0.58) | 19 (0.48) | 12 (0.54) | 25 (0.60) | 47 (0.63) |
| 65-74 years (N, %) | 123 (0.69) | 30 (0.76) | 11 (0.50) | 35 (0.84) | 47 (0.63) |
| 75-84 years (N, %) | 62 (0.35) | 26 (0.66) | — | 10 (0.24) | 21 (0.28) |
| ≥85 years (N, %) | 27 (0.15) | 17 (0.43) | — | — | — |
| Suppressed or age not stated (N, %) | 11 (0.06) | 13 (0.33) | 17 (0.77) | 17 (0.41) | 18 (0.24) |
| **Neoplasms in situ, benign, or of uncertain or unknown behavior** | | | | | |
| **Overall (N, %)** | 67 (0.38) | 17 (0.43) | 12 (0.54) | 13 (0.31) | 25 (0.33) |
| **Mean age of death (years, SD)** | — | — | — | — | — |
| **Sex** |  |  |  |  |  |
| Female (N, %) | 26 (0.15) | — | — | — | — |
| Male (N, %) | 41 (0.23) | — | — | — | 17 (0.23) |
| Suppressed or sex not stated (N, %) | 0 (0.00) | 17 (0.43) | 12 (0.54) | 13 (0.31) | 8 (0.11) |
| **Age** |  |  |  |  |  |
| <35 years (N, %) | — | — | — | — | — |
| 35-44 years (N, %) | — | — | — | — | — |
| 45-54 years (N, %) | — | — | — | — | — |
| 55-64 years (N, %) | 13 (0.07) | — | — | — | — |
| 65-74 years (N, %) | 24 (0.13) | — | — | — | — |
| 75-84 years (N, %) | 15 (0.08) | — | — | — | — |
| ≥85 years (N, %) | — | — | — | — | — |
| Suppressed or age not stated (N, %) | 15 (0.08) | 17 (0.43) | 12 (0.54) | 13 (0.31) | 25 (0.33) |

**Note:** All percentages are calculated using total decedents in each racial/ethnic subgroup as the denominator

**^1^**NHPI, Native Hawaiian and Pacific Islander

— indicates suppressed data values
